# A Platform for High-throughput and Ultrasensitive Immunopeptidomics

**DOI:** 10.1016/j.mcpro.2026.101590

**Published:** 2026-05-18

**Authors:** Adillah Gul, Laura Van Moortel, Patrick Willems, Ilke Aernout, Laura Pedró-Cos, Kia C. Ferrell, Katie Boucher, An Staes, Simon Devos, Ine Lentacker, Bart Vandekerckhove, Caroline Demangel, Fabien Thery, Francis Impens

**Affiliations:** 1 VIB Center for Medical Biotechnology, VIB, Ghent, Belgium; 2 Department of Biomolecular Medicine, Ghent University, Ghent, Belgium; 3 Ghent Research Group on Nanomedicines, Ghent University, Ghent, Belgium; 4 Cancer Research Institute Ghent (CRIG), Ghent, Belgium; 5 Institut Pasteur, Université Paris Cité, Inserm U1224, Immunobiology and Therapy Unit, Paris, France; 6 VIB Proteomics Core, VIB, Ghent, Belgium; 7 Department of Diagnostic Sciences, Ghent University, Ghent, Belgium; 8 GMP Unit Cell Therapy, Ghent University Hospital, Ghent, Belgium

**Keywords:** immunopeptidomics, antigen discovery, *Listeria monocytogenes*, bacillus Calmette-Guérin, BCG, major histocompatibility complex, MHC, timsTOF

## Abstract

Mass spectrometry-based immunopeptidomics is a powerful approach for untargeted discovery of peptides presented on MHC molecules, which can guide the selection of vaccine antigens and immunotherapy targets. First-generation immunopeptidomics workflows require processing of hundreds of millions of cells using lengthy, manual procedures. More recent approaches focus on increasing either sensitivity or throughput, but rarely combine both aspects. Here, we describe a semiautomated immunopeptidomics platform that combines high sensitivity with high throughput by implementing highly optimized conditions for isolation of MHC class I and II peptides in a 96-well positive pressure device. Lysis in a small volume of 100 μl allows efficient MHC capture in a 96-well filter plate with optimal pore size for automated washing, elution, and C18 purification steps. Upon analysis of 25% of the eluate from 16 million cells, our workflow identified over 13,500 MHC I and 6000 MHC II peptides on a timsTOF single-cell proteomics mass spectrometer, operating in data-dependent acquisition-parallel accumulation–serial fragmentation mode. Exploring the sensitivity limits of our platform, we identified up to 1000 MHC I peptides, including hundreds of predicted binders, from as few as 20,000 JY cells. Validating the platform’s performance for quantitative biological discovery, we report the identification of known and novel bacterial immunopeptides from U937 macrophages infected with *Listeria monocytogenes* or bacillus Calmette-Guérin. Together, our optimized immunopeptidomics platform enables robust immunopeptide detection from lower-input samples in a high-throughput fashion, enabling its use for biological applications where sample amounts are limiting.

## Highlights

• A semiautomated 96-well platform for low-input isolation of MHC-I and -II peptides.
• Identification of hundreds of MHC-I peptides from 20,000 cells.
• Quantitative detection of bacterial immunopeptides from infected macrophages.

## Graphical Abstract

**Figure:**
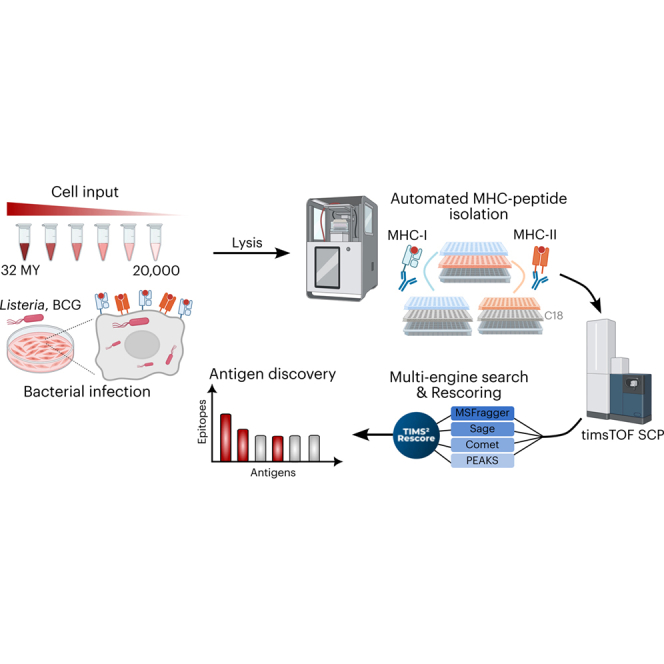

## In Brief

Immunopeptidomics enables untargeted discovery of peptides presented by MHC molecules, informing vaccine and immunotherapy development. Existing workflows focus either on throughput or sensitivity, while ideally both are combined. We describe a semiautomated, 96-well platform for high throughput MHC class I and II peptide isolation from low-input samples. The workflow identifies hundreds of MHC peptides from as few as 20,000 cells and successfully detects bacterial immunopeptides from *Listeria*- and BCG-infected macrophages, enabling biological applications where sample availability is limited.

The immunopeptidome is the repertoire of peptides presented by major histocompatibility complex (MHC) molecules, also known as human leukocyte antigens (HLAs) in humans, on the surface of cells (1). These MHC-bound peptides, called immunopeptides, are derived from self and nonself proteins processed inside the cell (2). Human MHC class I molecules present peptides that are 8- to 12-amino acids in length that result from proteasomal degradation of intracellular proteins. MHC class II molecules typically present 13- to 18-mer peptides generated by the uptake and lysosomal degradation of extracellular proteins. Peptides presented on MHC-I and MHC-II are recognized by CD8^+^ and CD4^+^ T cells, respectively. Therefore, the immunopeptidome plays a critical role in adaptive immunity, facilitating recognition of peptides derived from pathogens, tumor antigens, and aberrantly expressed self-proteins by T cells (3, 4, 5, 6). Over the last decade, mass spectrometry (MS)-based immunopeptidomics has emerged as a powerful analytical tool for comprehensive profiling of the immunopeptidome (2, 7), enabling the discovery of bacterial antigens presented during infection (8, 9, 10, 11, 12, 13, 14, 15, 16) or tumor-specific epitopes that can be used for immunotherapy (17, 18, 19, 20, 21), among other applications.

A typical MS-based immunopeptidomics workflow consists of three steps. It starts with sample preparation, which comprises the pulldown of MHC-peptide complexes and elution of purified immunopeptides. This is followed by LC-MS/MS analysis, where mass spectra of the intact and fragmented immunopeptides are recorded. Finally, the acquired MS data is searched by dedicated software tools to assign spectra to peptide sequences (22, 23, 24, 25). Although all three steps influence the quality of the final results, robust sample preparation is key as it directly influences the net peptide yield and overall reproducibility. This step is the most time-consuming due to the complexity of immunopeptide isolation and is often considered a major bottleneck for the clinical implementation of immunopeptidomics pipelines (26). Sample preparation begins with mild lysis of cells or tissues to extract MHC-peptide complexes in their native state. Immunoprecipitation (IP) is the preferred method to isolate these complexes, using specific antibodies for MHC-I and MHC-II. Once isolated, immunopeptides are eluted from the MHC molecules under mild acidic conditions, followed by a desalting step to purify the peptides for LC-MS/MS analysis (22, 27, 28). An alternative, antibody-free approach for MHC-I peptide isolation is mild acid elution (MAE) directly from the cell surface (29, 30, 31).

In recent years, technological advances have revolutionized the field of immunopeptidomics, allowing high-throughput extraction and analysis of immunopeptides. Innovations in MS instrumentation and data acquisition have greatly improved the sensitivity of immunopeptidomics workflows. This includes for instance trapped ion mobility spectrometry (TIMS) (28, 32, 33), data-independent acquisition (29, 34, 35, 36), and advanced data analysis by incorporating multiple search engines and/or data-driven rescoring (32, 33, 37, 38). Consequently, this has facilitated the evolution of sample preparation to higher throughput workflows on lower input samples. Initial sample preparation workflows, despite showing excellent peptide enrichment, required hundreds of millions of cells as starting material and depended on manual IP of immunopeptides in tubes or columns (15, 22, 25, 39). These workflows were laborious, time-consuming, and prone to variability, limiting their utility for large sample sets or scarce primary specimens. Since then, IP procedures have significantly improved and upgraded from individual column with large sample amounts to multi-well plate format compatible with lower input (26, 40, 41, 42). Together with increased instrumental and computational sensitivity, such miniaturized sample preparation workflows have overcome the challenge of performing immunopeptidomics on samples with limited material such as scarce tumor sections, lymph node biopsies, and rare cell populations.

At the same time, automation in sample preparation has remarkably enhanced overall throughput, reproducibility and sensitivity by reducing sample loss and contamination risks (26, 28, 40, 41, 42, 43, 44, 45, 46). The use of 96-well plates in immunopeptidomics sample preparation was first introduced by Chong *et al.* (26) in 2018, using stacked 96-well filter plates in combination with a positive pressure device for sequential MHC-I and MHC-II IP on tissue and cell lysates (15, 25). Since then, several other advancements in immunopeptidomics sample preparation have been reported (19, 27, 29, 30, 31, 32), focusing on further optimizing throughput in 96-well plate workflows (28, 44) or maximizing sensitivity for low-input samples, for instance, using microfluidics (42, 43, 45, 46). However, most workflows today focus on either throughput or sensitivity, while ideally, the combination of both should be achieved. To fill this gap, we have developed a refined sample preparation workflow that is fully optimized to process low-input and crude samples in 96-well plates, aiming to combine high throughput with maximal sensitivity. We implemented a miniaturized and automated workflow in a 96-well format using a Tecan Resolvex A200 liquid handling positive pressure device. Different from previous 96-well procedures, MHC pulldown occurs in a small lysate volume of 100 μl under static conditions, directly in a 96-well filter plate with optimized pore size. We demonstrate sensitive detection of immunopeptidomics from 32 million to 0.5 million human cells, with the ability to recover nearly 300 predicted MHC-I binders from just 20,000 JY cells. We showcase how this miniaturized platform facilitates bacterial antigen discovery for *Listeria monocytogenes* and the tuberculosis vaccine strain bacillus Calmette-Guérin (BCG) in a quantitative manner.

## Experimental Procedures

### Cell Lines

The Epstein–Barr virus-immortalized human B cell line JY (ECACC 94022533), human HeLa cells (ECACC 93021013), and human U937 (ATCC) cells were cultured at 37 °C in a humidified atmosphere at 5% CO_2_. JY and U937 cells were maintained in RPMI 1640 medium with GlutaMax (#61870036, Thermo Fisher Scientific), supplemented with 10% heat-inactivated fetal bovine serum (FBS, #10270106, Thermo Fisher Scientific) without antibiotics. U937 cells were differentiated using phorbol 12-myristate 13-acetate (PMA) at 200 nM final concentration for 48 h. HeLa cells were grown without antibiotics in DMEM high glucose medium with GlutaMax and pyruvate (#12077549, Thermo Fisher Scientific) supplemented with 10% FBS. All the cells were tested negative for *mycoplasma* contamination. Cells were counted and volumes corresponding to the number of cells in the serial dilution experiment were collected by centrifugation at 300 × *g*, washed twice with ice-cold PBS and stored at −80 °C.

### Bacterial Culture

A defrosted aliquot of BCG Pasteur strain was grown in Difco Middlebrook 7H9 broth Medium (#271310, Becton Dickinson) supplemented with 10% oleic albumin dextrose catalase, 0.2% glycerol, and 0.05% Tween 80 (Sigma-Aldrich, Merck), shaking at 37 °C. *L. monocytogenes* EGD (BUG600 strain) was grown in brain heart infusion broth (#10462498, Thermo Fisher Scientific), shaking at 37 °C. All the BCG and *Listeria* work was carried out aseptically in a biosafety level 2 facility.

### BCG and *Listeria* Infection of U937 Cells

U937 cells were differentiated into phagocytic macrophage-like cells by stimulation with 200 nM PMA for 48 h, followed by 24 h recovery in PMA-free media. BCG was cultured into the log phase and pelleted by centrifugation at 3900 × *g* for 7 min. Bacteria were washed once with PBS, resuspended in PBS, and filtered through 25, 26, and 27 gauge needles to acquire a single-cell suspension. Differentiated U937 cells were infected at a multiplicity of infection of 20 for 3 h, and the medium was aspirated to remove extracellular BCG and after which fresh media was added. Infected and uninfected cells were harvested with a cell scraper after 24 h, washed two times with PBS, and dry cell pellets were stored at −80 °C.

*Listeria* was grown overnight and diluted in brain heart infusion broth to a density of 1e9 bacteria/ml, washed twice with PBS and resuspended in U937 growth medium without FBS. U937 cells were washed two times with PBS prior to 1 h infection with *Listeria* at an multiplicity of infection of 25. Following infection, the cells were washed twice with PBS and further cultured for 23 h in cell culture medium supplemented with 10% FBS and 40 μg/ml gentamicin (#G1397, Sigma-Aldrich, Merck) to eliminate extracellular bacteria. Cells were harvested using a cell scraper, washed two times with PBS, and dry cell pellets were stored at −80 °C.

### Generation of Immunoaffinity Columns for MHC Class I and II Pulldown

W6/32 monoclonal pan-MHC class I and PdV5.2 monoclonal pan-MHC class II antibodies were purified from HB95 (HB-95, ATCC) and PdV5.2 (kindly provided by Bart Vandekerckhove, Ghent University (47)) hybridoma cells, respectively. To prepare the W6/32 immunoaffinity column, 3 mg of antibody was diluted in 5 ml tris-buffered saline and added to 1 ml pre-washed protein-A Sepharose 4B packed beads (#101041, Thermo Fisher Scientific) in glass Econo-columns (#7374150, Bio-Rad). To prepare the PdV5.2 immunity affinity column, 1.5 mg of antibody was diluted in 5 ml tris-buffered saline and added to protein-G Sepharose 4B packed beads (#101242, Invitrogen). Antibodies were incubated at room temperature for 1 h on an Ika rolling tube mixer device. Each column was washed with 0.2 M sodium tetraborate buffer (pH 9, #B3545, Sigma-Aldrich, Merck) followed by 40 min chemical crosslinking using 20 mM dimethylpimelimidate (#D8388, Sigma-Aldrich, Merck), freshly dissolved in sodium tetraborate buffer. After crosslinking, antibody-bead complexes were washed and incubated for 2 h on an Ika mixer with 5 mL 0.2 M ethanolamine (pH 8, #149582500, Thermo Fisher Scientific) to quench the crosslinking reaction. A more detailed description of the procedure can be found in the Supplemental Methods.

### High-throughput Isolation and Purification of MHC-I and MHC-II Peptides

#### Cell Lysis

Cell pellets ranging from 0.5 to 32 million cells were lysed in 0.1 ml mild lysis buffer containing 1% octyl β- D-glucopyranoside (#O9882, Sigma-Aldrich, Merck), 0.25% sodium deoxycholate (#1065040250, Millipore, Merck), 1.25 × cOmplete protease inhibitor cocktail (#4693159001, Roche), 1 mM PMSF (#52332, Sigma-Aldrich, Merck), 0.2 mM iodoacetamide (#I1149, Sigma-Aldrich, Merck), and 1 mM EDTA (#EDS, Sigma-Aldrich, Merck) in 150 mM NaCl, 50 mM Tris-HCl (pH 8.0). Cells were lysed for 1 h on ice while resuspending every 15 min, then centrifuged at 20,000 × *g* at 4 °C for 10 min to remove cell debris, followed by a second centrifugation at 20,000 × *g* at 4 °C for 30 min to further clear the lysate.

#### Preparation of Filter Plates for MHC-I and II Pulldown

We utilized a Resolvex A200 positive pressure processor (Tecan) to automate the washing and elution steps. Subsequent IPs of MHC-I and MHC-II complexes were performed using a single-use 96-well filter microplate (0.7 μm glass fiber membrane, 2 ml/well, long drip Agilent [part #201719-100]). Approximately 5 to 10% low positive pressure was applied at each step to maintain a uniform liquid flow through the plates. A more detailed description of the procedure can be found in the Supplemental Methods.

**Fig. 1:**
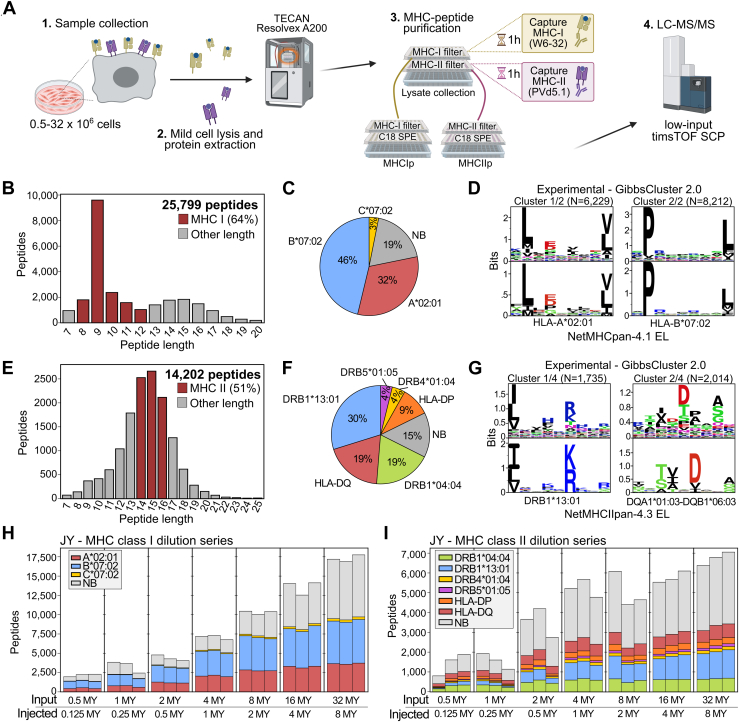
**A sensitivesemiautomated workflow for high-throughput immunopeptidomics.***A*, general overview of the workflow. Cells are lysed in mild conditions, after which proteins are extracted and loaded on 96-well plates with pre-washed anti-MHC immunoaffinity beads. Sequential MHC-I and MHC-II IP, elution and desalting are performed in a semiautomated fashion on a Resolvex A200 (Tecan), after which peptides are collected and 25% is injected for LC-MS/MS on a timsTOF SCP instrument. Figure created with BioRender.com. *B*, histogram showing the length distribution of peptides identified after MHC class I pulldown (peptide Q-value ≤1%). Peptides compatible with MHC-I binding (length 8–12) are indicated in *red*, nonbinding (NB) peptides in *gray*. *C*, proportion of identified 8 to 12 mers predicted to bind to the indicated HLA alleles by NetMHCpan-4.1 (53). *D*, unsupervised Gibbs clustering (55) of identified 8 to 12 mers resulting in sequence logos that match MHC class I eluted ligand (EL) motifs of NetMHCpan-4.1 (53). *E*, histogram showing the length distribution of peptides identified after MHC class II pulldown and elution. Peptides compatible with MHC-II binding (length 14–16) are indicated in *red*, NB peptides in *gray*. *F*, proportions of identified 14 to 16 mers predicted to bind to the indicated HLA alleles by NetMHCIIpan-4.3 (54). For HLA-DQ and HLA-DP, the possible α–β chain pairings (*e.g.* HLA-DQA10103-DQB10603) were grouped together. *G*, unsupervised Gibbs clustering (55) of identified 14 to 16 mers resulted in sequence logos that match MHC class II eluted ligand (EL) motifs of NetMHCIIpan-4.3 (54). *H* and *I*, number of identified peptide sequences for individual replicates, grouped by cell input amount. Samples from each amount were processed and searched separately for these graphs. Peptides are colored according to binding prediction for JY MHC class I alleles (*H*) and class II alleles (*I*), and NB peptides in *gray*. HLA, human leukocyte antigen; IP, immunoprecipitation; MS, mass spectrometry; NB, nonbinding; SCP, single-cell proteomics.

Before IP, each well was activated with 1 ml 100% acetonitrile (ACN) (Sigma-Aldrich) twice, followed by three washes with 1 ml 0.1% TFA and finally three times with 1 ml 150 mM NaCl 50 mM Tris-HCl (pH 8.0). Next, the MHC-I and MHC-II crosslinked beads were loaded on their respective plates (further referred to as “MHC-I filter” and “MHC-II filter” plates, Fig. 1*A*). Beads were washed five times with 1 ml 150 mM NaCl 50 mM Tris-HCl (pH 8.0).

#### Subsequent MHC-I and MHC-II IP

Lysates were added to the MHC-I filter plate and incubated for 1 h at 4 °C to capture MHC-I complexes. Next, the unbound lysate fraction of the MHC-I IP was collected using the Tecan and loaded on the MHC-II filter plate, followed by 1 h incubation at 4 °C. Meanwhile, the MHC-I filter plate was washed five times with 1 ml 150 mM NaCl in 50 mM Tris-HCl (pH 8.0) using the Tecan. After 1 h incubation, the MHC-II filter plate was washed similarly.

#### Immunopeptide Desalting and Elution

Sep-Pak tC18 plates (#186002321, Waters) were activated with two times 1 ml 100% ACN and three times 1 ml Milli-Q water containing 0.1% TFA, using approximately 1 to 3% high positive pressure on the Resolvex A200 to maintain a uniform liquid flow throughout the plates. Each MHC filter plate was stacked on top of a Sep-Pak plate (Fig. 1*A*) and MHC-peptide complexes were eluted with 0.2 ml 10% acetic acid five times. Next, we removed the filter plate and washed the Sep-Pak plate three times with 1 ml of 0.1% TFA. Thereafter, a collection plate (#201240-100, Agilent) was mounted under the Sep-Pak tC18 plate. MHC-I peptides were eluted three times with 25% ACN/0.1% TFA, while MHC-II peptides were eluted with 40% ACN/0.1% TFA. The eluted immunopeptides were then transferred to protein LoBind tubes (#0030108450, Eppendorf), vacuum dried and stored at −20 °C until LC-MS/MS injection.

### LC-MS/MS Analysis

**Fig. 2:**
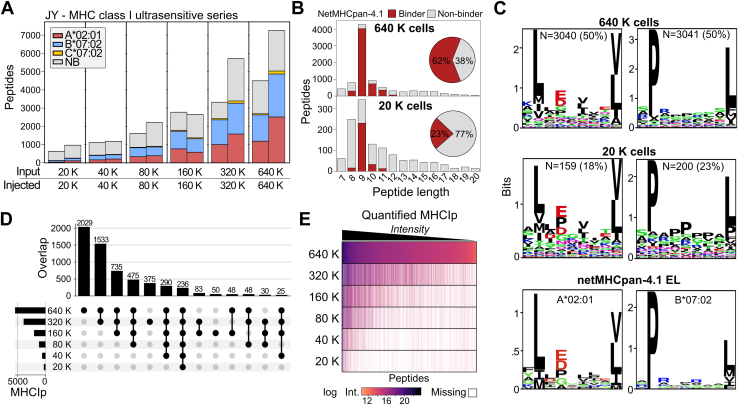
**Toward ultrasensitive immunopeptidomics.***A*, number of identified peptide sequences per replicate, grouped per cell input amount from 640,000 (640 K) down to 20,000 cells (20 K). Samples from each input amount were processed and searched independently, and 100% of the isolated immunopeptides were analyzed by LC/MS-MS. Peptides are colored according to MHC binding prediction by NetMHCpan-4.1 (53). *B*, histogram showing the length distribution of peptides identified after MHC class I pulldown and elution from 640 K and 20 K cells. Peptides compatible with MHC-I binding (length 8–12) are indicated in *red*. *C*, unsupervised Gibbs clustering (55) of identified class I peptides from 640 K and 20 K cells (*top and middle*) resulted in sequence logos that match NetMHCpan-4.1 eluted ligand (EL) sequence logos for HLA-A∗02:01 and HLA-B∗07:02 (*bottom*). *D*, UpSet plot showing the overlap of identified predicted MHC-I peptides (MHCIp, NetMHCpan-4.1 %Rank < 2) across the different input amounts. Only intersections of more than 10 peptides are displayed. *E*, heatmap showing log_2_ intensities of all peptides identified in the 640 K dilution, sorting peptides from highest to lowest intensity in this dilution (*left* to *right*). Missing values are colored in *white*. Cell input amounts were searched separately using the FragPipe “Nonspecific-HLA” workflow. HLA, human leukocyte antigen; MS, mass spectrometry.

Dried peptides were dissolved in 50 μl 0.1% ACN/0.1% TFA from which 12.5 μl was injected for LC-MS/MS analysis on a Ultimate 3000 RSLC nanoLC in-line connected to a timsTOF single-cell proteomics (SCP) mass spectrometer (Bruker). Of note, the dried peptides from low input dilution series (Fig. 2) were dissolved in 17 μl 0.1% ACN/0.1% TFA from which 15 μl was injected for LC-MS/MS analysis. Trapping was conducted at a flow rate of 20 μl/min for 2 min in loading solvent A on a 5 mm trapping column (Thermo Fisher Scientific, Pepmap, 300 μm internal diameter [I.D.], 5 μm beads). The sample was then separated on a reverse-phase column (Aurora elite 75 μm × 150 mm 1.7 μm particles, IonOpticks) following elution from the trapping column. Separation was achieved using a linear gradient, starting at 0.5% MS solvent B (0.1% FA in water/ACN 20:80, v/v) at 250 nl/min for 30 min, increasing to 37.5% MS solvent B, then reaching 55% at 38 min, and further rising to 70% at 40 min. This was followed by a wash for 5 min and reequilibration with 99.5% MS solvent A (0.1% FA in water). The flow rate was adjusted from 250 nl/min to 100 nl/min at 20 min and restored to 250 nl/min at 40 min. A 10 parallel accumulation–serial fragmentation (PASEF)/MSMS scan acquisition method was employed in data-dependent acquisition (DDA)-PASEF mode with a precursor signals intensity threshold of 500 arbitrary units. Precursors were isolated with a 2 Th window below *m/z* 700 and 3 Th above, with active exclusion for 0.4 min upon reaching a target intensity threshold of 20,000 arbitrary units. In case of MHC-I peptides, an extended IM range (0.75–1.65 Vs/cm^2^) was applied to include HLA-I singly charged precursors, whereas a more narrow IM range (range 0.7–1.25 Vs/cm^2^) was used for MHC-II peptides (Supplemental Fig. S1). The mass spectrometer was operated in nonsensitive mode with an accumulation and ramp time of 100 ms, analyzing in MS from 100 to 1700 *m/*z, applying collision energy according to the IM range specified in Supplemental Table S1.

### Data Analysis and Processing

#### Immunopeptidomics Searches and Analysis

Raw proteomics data were searched against the human reference proteome (August 2024, UP00005640, 20,666 proteins) using a multi-search engine workflow with follow-up rescoring as described in Willems *et al.* (33). In case of U937 infected cell experiments, *L. monocytogenes* strain EGD (UP000016703, 2847 proteins) and *Mycobacterium bovis* strain BCG/Pasteur (UP000001472, 3891 proteins) proteomes were appended to the human proteome for database searching. In brief, timsTOF (.d) data was searched by four search engines (MSFragger (48), Comet (49), Sage (50), and PEAKS Studio 12 (51)) after which search results for each engine were rescored in parallel using TIMS^2^Rescore (52). Peptides identified below a 1% peptide q-value were reconciled at the spectrum-level, excluding the rare cases where a spectrum was matched to distinct peptides by different search engines (33). Downstream immuno-informatic analyses included NetMHCpan-4.1 (53) and NetMHCIIpan-4.3 (54) prediction of binding strength for 8–12-mers and 14–16-mers, respectively, and unsupervised Gibbs clustering (55). Default NetMHCpan %Rank score thresholds were used to define strong and weak MHC class I binders (below 0.5 and 2, respectively) and MHC class II binders (below 2 and 5, respectively).

#### Differential Analysis

To analyze differential immunopeptide abundances between infected and uninfected samples, we used FragPipe (version 22.0) including MSBooster (38) and IonQuant quantification (56). Resulting peptide intensities (from “combined peptides.tsv” reports) were used for peptide-level differential statistics. To assess differential peptide abundance, we resorted to an empirical Bayes test in limma (57) following recommendations of a statistical benchmark (58). Significantly regulated peptides were filtered at an adjusted *p* value ≤ 0.05 and absolute fold change ≥ 2. This differential test does not impute missing values, with the drawback of not reporting peptides exclusively detected in one of the conditions (infected/uninfected). Therefore, we performed an additional differential detection analysis where we discerned peptides/proteins uniquely identified in one condition (three out of four replicates) and absent in the other condition.

#### Gene Set Enrichment Analysis

For the differential immunopeptides identified during infection, their corresponding UniProtKB accessions were used for protein-level gene set enrichment analysis. In the case a peptide matched multiple proteins, the first UniProtKB accession was used as representative. Accessions were entered in the online webtool g:Profiler using default settings (59).

#### Visualization

Plots were generated using matplotlib (60) and seaborn (61) in Python (version 3.8.10). Sequence logos were generated using Logomaker (62). UpSet plots were made using the Python implementation module *upsetplot* (63).

### Experimental Design and Statistical Rationale

**Fig. 3:**
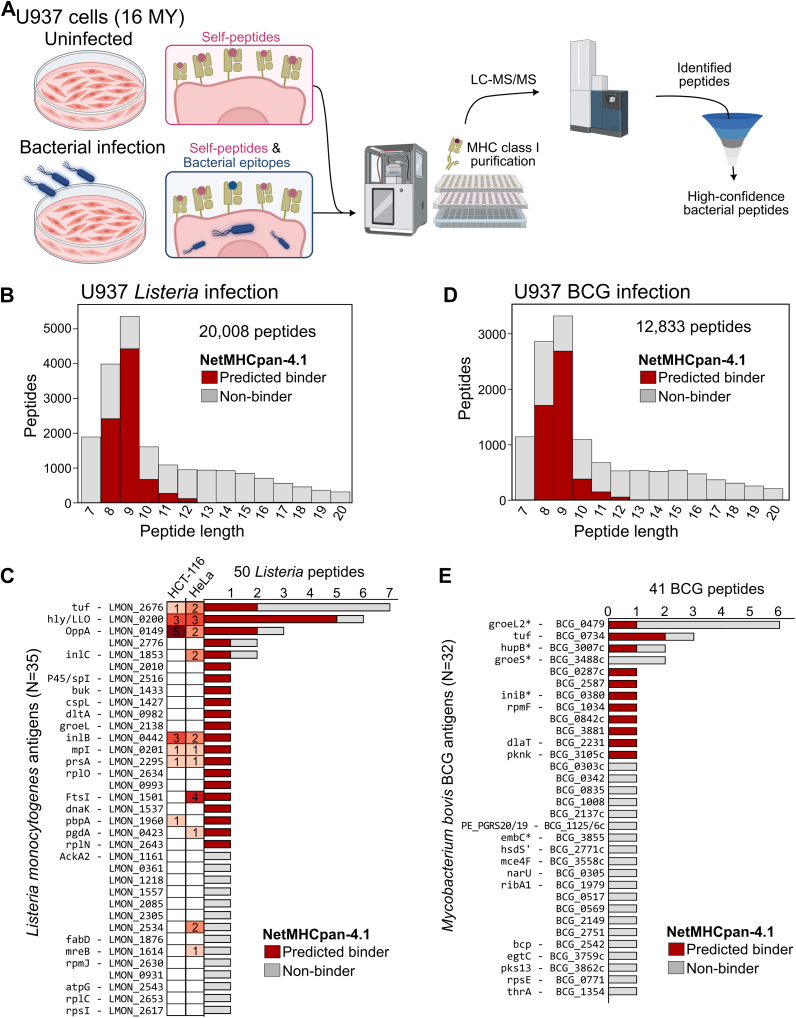
**Bacterial antigen detection.***A*, bacterial immunopeptidomics workflow scheme. Differentiated U937 cells infected with bacteria and uninfected counterparts are subjected to the immunopeptidomics workflow (16 million cell input, 25% injected). This enables the detection of bacterial epitopes among numerous host self-peptides. After purification and LC-MS/MS of immunopeptides, data was filtered to retain only high-confidence bacterial immunopeptides. Figure created with BioRender.com. *B* and *D*, histogram showing the length distribution of peptides identified after MHC class I pulldown. Peptides predicted to bind to U937 MHC-I alleles by NetMHCpan-4.1 (53) (%Rank < 2) are indicated in *red*. *C* and *E*, the number of unique immunopeptides identified per *Listeria* protein (*C*) or *Mycobacterium bovis* BCG protein (*E*) is shown in a histogram. Immunopeptides predicted as MHC class I binders by NetMHCpan-4.1 (53) (%Rank < 2) are indicated in *red*, other peptides in *gray*. *C*, the number of identified peptides per cell line in our previous *Listeria* study (8), which used 350 to 540 million cells as input per replicate, is displayed in a heatmap. *E*, BCG proteins identified in previous immunopeptidomics studies (9, 10, 68, 69) are indicated by an asterisk (∗). BCG, bacillus Calmette-Guérin; MS, mass spectrometry.

**Fig. 4:**
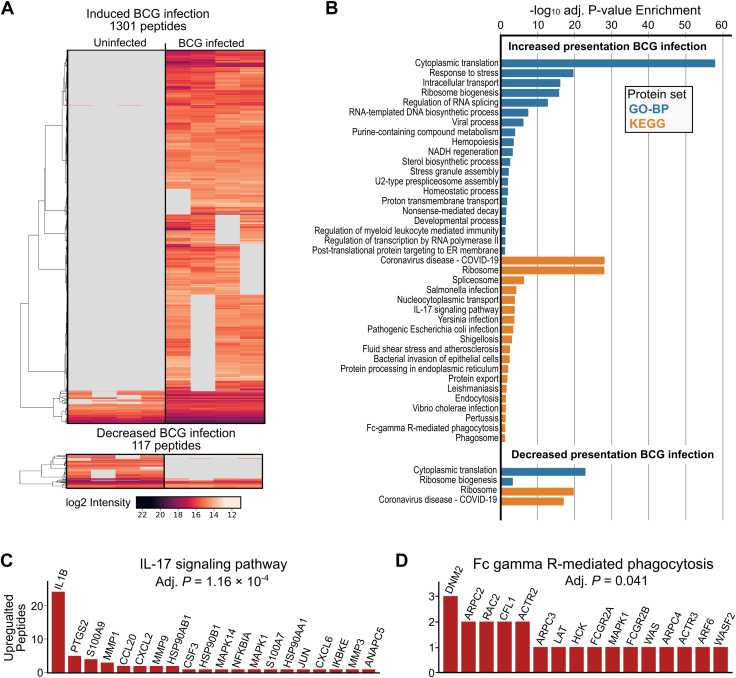
**Host immunopeptide remodeling in response to BCG infection.***A*, heatmap showing log 2 intensities of significant differentially regulated immunopeptides (adj. *p* value ≤ 0.05 and absolute fold change ≥ 2) as well as immunopeptides uniquely detected in three out four replicates in infected or uninfected condition. *B*, functional enrichment analysis of UniProtKB protein accessions with up- or downregulated immunopeptides under infection using g:Profiler (59). *C* and *D*, number of significantly upregulated peptides in the enriched protein sets “IL-17 signaling pathway” (*C*) and FcγR-mediated phagocytosis (*D*). BCG, bacillus Calmette-Guérin.

Immunopeptidomics optimization experiments (Supplemental Figs. S2–S6) were conducted in technical IP replicates, with a single 25% injection of each sample on a timsTOF SCP instrument. Immunopeptidomics benchmarking experiments using JY, HeLa, and U937 cells (Fig. 1 and Supplemental Figs. S7–S11) were performed with biological triplicates, starting from individual cell pellets for each replicate and cell number. 25% of each sample was injected once on the timsTOF SCP, going from low to high input to minimize sample carry-over. Ultrasensitive immunopeptidomics benchmarking experiments using JY cells (Fig. 2 and Supplemental Figs. S12–S16) were performed in two biological replicates, starting from individual cell pellets for each replicate and each cell number. 100% of each sample was injected on the timsTOF SCP instrument, going from low to high input. *L. monocytogenes* infection experiments were performed in three technical replicates (Fig. 3 and Supplemental Fig. S17), while BCG infection experiments were conducted in four biological replicates (Fig. 3 and 4 and Supplemental Figs. S17–18). For these experiments, 25% of each sample was injected once on the timsTOF SCP using a randomized block design, and significantly regulated peptides were defined at an adjusted *p* value ≤ 0.05 and absolute fold change ≥2 using an empirical Bayes test in limma (57) following recommendations of a statistical benchmark (58). Also peptides reproducibly detected in three out of four biological replicates, but absent in the other condition were defined as significantly regulated.

## Results

### An Automated Workflow for High-throughput Immunopeptidomics

Our previous immunopeptidomics workflow required approximately 500 million cells as input for manual IP in large columns (8, 15), thus requiring large sample input amounts with limited throughput. Here, we aimed to miniaturize and automate sample preparation by reducing the input amount 10- to 50-fold while increasing sample throughput by using a 96-well format. We initially tested optimal conditions for low-input cell lysis, IP and elution on JY cells, an Epstein–Barr virus-immortalized human B cell line, and found that static IP at high protein concentration (small lysate volume) with elution in 10% acetic acid led to the highest number of identified MHC-I and II peptides (Supplemental Figs. S2–S5). This optimized procedure was then automated on a Tecan Resolvex A200 positive pressure device, using 96-well filter plates with a pore size of 0.7 μm preventing flow-through by gravity, but allowing a controlled and consistent flow through the wells when positive pressure is applied. In the resulting workflow, cell pellets are lysed in 100 μl and cleared lysates are loaded on 96-well filter plates for sequential pulldown of MHC-I and II complexes, directly on the plate under static conditions. Following MHC capture, the flow-through is pushed through and multiple wash steps are applied by automated buffer dispensing by the Tecan. Immunopeptides are eluted under acidic conditions and collected in a 96-well Sep-Pak C18 plate for further purification, also on the Tecan (Fig. 1*A*). Performing MHC-II after MHC-I pulldown was necessary to avoid coenrichment of MHC-I peptide complexes during MHC-II IP (Supplemental Fig. S6). We also found that the pan-MHC-II PdV5.2 antibody shows similar performance to other MHC class II antibodies (TU39 or IVA12, Supplemental Fig. S6). Finally, isolated immunopeptides are analyzed by LC-MS/MS analysis using a timsTOF SCP mass spectrometer operating in DDA-PASEF mode, followed by immunopeptide identification using four different search engines with data-driven rescoring as described (33).

To test the sensitivity of the immunopeptidomics platform, we enriched MHC class I and II peptides from JY cells, using input amounts ranging from 0.5 to 32 million cells. For each input amount, triplicate samples were processed side by side, and from each sample, 25% of the isolated immunopeptides was injected onto the timsTOF SCP, thus corresponding to 0.125 to 8 million cell equivalents injected. First, we assessed the global quality of the immunopeptidome by searching all samples together. Over all input amounts, a total of 25,799 peptides were identified in the MHC-I pulldown, including 16,404 (64%) 8- to 12-mers (Fig. 1*B*, Supplemental Data S1). The majority of these 8- to 12-mers (81%) were predicted to bind at least one JY HLA allele (Fig. 1*C*) and clustered according to the anticipated HLA binding motifs (Fig. 1*D*). For MHC-II, we identified 14,202 peptides in JY cells, including 7304 (51%) 14- to 16-mers (Fig. 1*E*,Supplemental Data S2), of which 85% were predicted binders (Fig. 1*F*) and yielded the expected MHC sequence motifs (Fig. 1*G*).

Next, we searched the samples from each input amount separately for fair comparison. For both MHC class I and II pulldowns, we observed a progressive decline in the number of identified peptides with decreasing input amount (Fig. 1, *H* and *I*). For instance, from 0.5 million cells (0.125 million injected) on average 2158 MHC-I peptides were identified, while from 32 million cells (8 million injected) on average 17,266 peptides were identified, approximately eight times more. Both the usage of multiple search engines and data-driven rescoring boosted immunopeptide identification, particularly at lower cell inputs (Supplemental Fig. S7). Approximately 50 to 60% of MHC-I peptides and 30 to 50% of MHC-II peptides were identified in all replicates in ≥2 million cell inputs (0.5 million injected), with MHC-I peptide intensities showing ∼0.9 pairwise Pearson correlation between replicates (Supplemental Figs. S8 and S9), demonstrating quantitative reproducibility of the workflow. Interestingly, the proportion of NetMHCpan-predicted MHC-I binders is slightly higher at lower input numbers. With 0.5 million cells (0.125 million injected) as input, 66% of the peptides were predicted to bind JY HLA alleles on average, while for 32 million cells (8 million injected), 55% were predicted binders. Closer inspection revealed this to be partially explained by reported unspecific coisolation of MHC-II binders (64) particularly at higher cell input amounts where MHC-II binders represent up to ∼50% of the nonbinding MHC-I peptides (Supplemental Data S1). As such, an increase in cell number input is not linearly correlated with the number of identified MHC class I-binding peptides. This is exemplified by the fact that the number of predicted MHC class I binders starts to plateau around 16 million cells (4 million injected) (Fig. 1*H*), while relative gains in MHC class II peptide identifications decrease around 4 million cells (1 million injected) (Fig. 1*J*). Using fixed and rather high amounts of antibody across all input amounts (300 μg W6/32 and 150 μg PdV5.2), likely these plateaus reflect a saturation in cell lysis or MHC solubilization rather than antibody capture. We also observed that for both MHC-I and -II peptides, the median binding strength increased with decreasing input amounts, indicating that the identification of strong-binding peptides is favored over weak-binding peptides in low-input samples (Supplemental Fig. S10).

To test whether these observations generalize beyond JY cells, we also isolated MHC-I peptides from different amounts of HeLa and U937 cells, representing human epithelial and macrophage cells, respectively. The detected peptides were also of high quality and showed preferred binding patterns to the anticipated HLA alleles, with a higher proportion of 8-mers in U937 cells due to immunopeptide presentation on HLA-B∗18:01 and HLA-B∗51:01 (Supplemental Fig. S11, *A*–*F*, Supplemental Data S3 and S4) (65). Although in general fewer peptides were identified compared to JY cells, for both cell types the number of identified immunopeptides also plateaued with a sample size of 16 million cells (4 million injected), which we therefore defined as the optimal input amount for maximum detection of immunopeptides (Fig. 1, *H* and *I*, Supplemental Fig. S11, *H* and *I*). Together, these experiments established an automated workflow for immunopeptidomics sample preparation in 96-well format, compatible with a wide range of input amounts.

### Toward Ultrasensitive Immunopeptidomics

Given the successful identification of more than 2000 MHC class I peptides from 0.5 million cells (0.125 million injected) (Fig. 1*H*), we tested the capability of our platform to identify immunopeptides from very low (< 1 million) cell input amounts. Starting from 0.64 million cells, we generated a two-fold serial dilution series down to 20,000 cells as input. The same automated sample preparation workflow was used, but now the full sample amount (100%) was injected on the timsTOF SCP. Analyzing both samples with 640,000 cells, we identified 8858 unique peptides of which 5482 (62%) were predicted MHC class I binders (Fig. 2, *A* and *B*, Supplemental Data S5). At a 30-fold lower input of only 20,000 cells, we still detected 1244 peptides with 292 (23%) predicted binders. A peak of 9-mers was still discernible, and unsupervised Gibbs clustering recovered the leading JY HLA sequence motifs of HLA-A∗02:01 and HLA-B∗07:02 (Fig. 2, *B* and *C*, Supplemental Fig. S12), indicating successful immunopeptide isolation at these ultra-low inputs. Also here, multi-engine rescoring aided in recovering peptides from these limited cell inputs (Supplemental Fig. S13). In addition, we repeated this experiment but now with application of the MS-compatible nonionic surfactant n-dodecyl-β-D-maltoside (DDM), which is used to increase peptide identification in single-cell experiments by decreasing peptide adsorption to plasticware (66). The same trend and number of identified peptides was observed without DDM, suggesting no beneficial effect of DDM in this setup (Supplemental Fig. S14, Supplemental Data S6).

Comparing identified MHC-binding peptides across input amounts revealed a common subset of class I peptides that progressively narrows with each dilution, eventually reaching 236 peptides that were identified across all dilutions (Fig. 2*D*). Thus, 236 out of 292 (81%) MHC-I-presented peptides in the 20,000-cell samples were present in all dilutions. To address why these particular immunopeptides were retained at very low input, we explored multiple biological and technical reasons. To explore the role of peptide intensity, we ran a FragPipe HLA peptidome analysis with IonQuant (56) quantification separately for each dilution (Supplemental Data S7). This revealed MS intensity to be a determining factor, reflecting high immunopeptide abundance and/or favorable MS ionization or LC elution, with high-abundant immunopeptides in the 640,000 cell input more likely to be identified in lower dilutions (Fig. 2*E*). The binding strength of predicted MHC-I binders was similarly distributed across dilutions (Supplemental Fig. S15). We also inspected the potential sources of the relatively higher proportion of predicted nonbinding (NB) peptides at very low cell dilutions. Although the origin of most of these peptides remains unclear, we observed that 25 to 30% of these peptides belong to typical proteomic contaminants (*e.g.* keratins) that were reproducibly identified across dilutions (Supplemental Fig. S16, *A* and *B*). NB peptides showed comparable search engine scores to predicted peptide binders, despite exhibiting lower intensities, suggesting these represent *bona fide* identifications present at lower abundance (Supplemental Fig. S16*C*). Column carryover was negligible, though a few dozen peptides identified in the 20,000 cell sample likely carried over from a preceding tryptic quality control and wash run (Supplemental Fig. S16*D*). Overall, this suggests NBs to originate from common MS contaminants introduced during low-input sample preparation in our setup.

### Detection of Bacterial Immunopeptides Presented by Infected Cells

When applied to infected cells or tissues, immunopeptidomics allows the untargeted detection of bacterial immunopeptides, making it a powerful technology for antigen discovery and bacterial vaccine development (8, 67). We previously reported the detection of bacterial immunopeptides presented by cells infected with *L. monocytogenes* (8, 15, 33, 36) using several hundred million cells as input. Here, we aimed to test whether our optimized workflow would achieve similar performance in bacterial antigen detection using lower cell input amounts. To this end, we infected 16 million U937 cell samples with *L. monocytogenes* EGD (further referred to as *Listeria*) and isolated MHC class I immunopeptides from three infected and three uninfected samples for LC-MS/MS analysis (Fig. 3*A*). In total, 20,008 peptides were identified, including 12,995 8- to 12-mers of which 7922 (61%) were predicted binders of U937 HLA alleles (Fig. 3*B*, Supplemental Data S8). The detected peptides showed preferred binding patterns to the anticipated HLA alleles, with again a higher proportion of 8-mers resulting from HLA-B∗18:01- and HLA-B∗51:01-presented peptides (Supplemental Fig. S17*A*). Next, we stringently filtered high-confidence bacterial peptides as described (33). Although most peptides were of human origin, we detected 50 high-confidence bacterial peptides derived from 35 *Listeria* proteins. Of these, elongation factor Tu (tuf), virulence factors hly/LLO and inlC, periplasmic oligopeptide-binding protein OppA and cell wall carboxypeptidase LMON_2776 were represented by multiple immunopeptides (Fig. 3*C*). This observation is in line with the previously reported immunodominance of these *Listeria* antigens, where tuf, hly/LLO and OppA were also identified by multiple immunopeptides in infected HeLa and HCT-116 cell cultures (8, 33, 36). In addition to *Listeria*, we applied our workflow to U937 cells infected with BCG. Here, 12,833 MHC class I peptides were identified, including 8480 8- to 12-mers with 4992 (59%) predicted binders (Fig. 3*D*, Supplemental Fig. S17*B*, Supplemental Data S9). We detected 41 high-confidence bacterial peptides derived from 32 BCG proteins. Again, several BCG proteins were represented by multiple immunopeptides, including elongation tuf, groEL2, groES and hupB (Fig. 3*E*). Interestingly, peptides from these three proteins were also identified by a previous immunopeptidomics study that mapped MHC class II peptides on BCG-infected cells (9, 10), and the same holds true for embC (68). Also IniB was identified in *Mycobacterium tuberculosis*-infected macrophages (69). Together, these data demonstrate the ability of our workflow to effectively capture relevant bacterial immunopeptides from limited amounts of infected cells.

### Quantitative Remodeling of the Human Immunopeptidome upon BCG Infection

Bacterial infection impacts many cellular processes, resulting in altered presentation of host cell immunopeptides in addition to bacterial peptides. Using a quantitative, nonspecific HLA workflow in FragPipe, we aimed to identify differential human immunopeptide presentation upon BCG infection. Firstly, we assessed the reproducibility of peptide quantification. Uninfected controls displayed a higher quantitative reproducibility (Pearson R = 0.885 ± 0.034) than BCG-infected samples (Pearson R = 0.794 ± 0.045), suggesting that infection introduces greater quantitative variability (Supplemental Fig. S18*A*). Furthermore, the multidimensional scaling plot based on immunopeptide intensities revealed a clear separation between uninfected controls and BCG-infected samples (Supplemental Fig. S18*B*). Next, we compared the intensities of the human immunopeptides between the BCG-infected and uninfected samples. This analysis revealed 1301 immunopeptides that were significantly upregulated (adj. *p* ≤ 0.05, ≥ two-fold change, 117 peptides) or uniquely present in the infected samples (1184 peptides), while 117 peptides were upregulated (32 peptides) or uniquely present in the uninfected samples (85 peptides) (Fig. 4*A*,Supplemental Data S10). Gene set enrichment analysis revealed that many regulated peptides (both up and down) were derived from ribosomal proteins, which in general are overrepresented MHC-I source proteins (45, 70, 71), and many peptides upregulated upon infection originated from proteins linked to infection, inflammation and immune signaling (Fig. 4*B*). For instance, interleukin-1 beta showed a drastic upregulation of 24 immunopeptides during infection, and was annotated alongside other immune-related proteins as part of the KEGG “IL-17 signaling pathway” that was significantly enriched (adj. *P* 1.16 × 10^−4^) (Fig. 4*C*). Interleukin-1 beta is a well-known pro-inflammatory cytokine that accumulates in monocytes and macrophages in response to pathogen infection (72, 73). Interestingly, it is produced in the cytosol as an inactive precursor protein that requires a maturation step for its release (73, 74), possibly facilitating its strong presentation by MHC-I under infection. In addition, proteins like Fcγ receptors (FCGR2A and B) and ARP2/3 complex subunits (ACTR and ARPC proteins) involved in “FcγR-mediated phagocytosis” were more presented by infected cells (adj. *P* 0.041) (Fig. 4*D*). Taken together, these data show the high reproducibility and quantitative performance of our workflow, revealing immune remodeling under infection, with increased presentation by MHC class I molecules of proteins involved in inflammation, phagocytosis, and other immune-related processes triggered by BCG infection.

## Discussion

We here describe a platform for immunopeptidomics sample preparation that is both sensitive and compatible with high-throughput analysis. Sequential capture of MHC class I and II immunopeptides is achieved through optimized IP in 96-well format on a positive pressure device. In combination with sensitive timsTOF SCP data acquisition and analysis (28, 32, 33) we demonstrate that the platform can comprehensively capture and identify MHC class I and II immunopeptidomes using sample sizes ranging from 32 to 0.5 million cells, with 16 million cells as optimum after which no further gain in detected binders was observed. Exploring the sensitivity limits of our platform, we could detect over 1200 peptides from only 20,000 cells, albeit challenged with lower percentages of predicted binders. To further showcase the discovery potential of the platform, we performed bacterial antigen discovery in 16 million U937 macrophages infected with *Listeria* or BCG. This led to the identification of known and novel bacterial antigens, despite a 30-fold reduction in input material compared to previous analyses (8, 9, 33, 36, 68, 69). Thus, our optimized platform allows sensitive immunopeptide detection in a high-throughput fashion, enabling its application on biological sample types where input amounts are limited.

Cell lysis, IP and elution steps are determining steps for successful immunopeptide isolation, which were all carefully optimized. Traditional IP protocols typically mix antibody-bound beads with lysate under agitation (8, 25); however, we observed that MHC-I IP under static conditions produces equivalent results (Supplemental Fig. S2, A–C). As a more important factor, we uncovered that efficient MHC-I IP heavily depends on a high protein concentration, which can be achieved by reducing the lysis buffer volume (Supplemental Fig. S2, D and E). To increase sample throughput, we tested multiple 96-well filter plates with pore sizes ranging from 25 to 0.2 μm using a positive pressure device. With larger pores causing leakage during incubation steps and smaller pores requiring higher pressure, we selected a 0.7 μm filter plate as an optimal middle ground, allowing a steady flow without leakage nor clogging. Finally, we tested IP elution and peptide purification methods (Supplemental Figs. S4 and S5), determining 10% acetic acid IP elution with Sep-Pak tC18 peptide purification as a preferred method.

Together, these optimizations led to the most important difference with previous multi-well sample preparation procedures (Supplemental Table S2): the small volume of only 100 µl used for lysis and MHC capture. Other multi-well procedures typically start from larger lysis volumes of 1 ml or higher such as the original 96-well procedure by Chong *et al.* (26). Inspired by this protocol, we also employ static IP, however, instead of relying on gravity flow, we optimized the plate pore size to prevent leakage and to allow flow control by positive pressure. Overall, our platform reduces sample transfers, thereby avoiding sample losses and enhancing sensitivity compared to procedures involving single tubes (8, 15) or incubation of deep well plates under rotation (28). Similar to other immunopeptidomics platforms using Waters (26), Agilent (40, 41) or KingFisher (42) devices, we implemented our procedure on a Tecan Resolvex A200 to automate the MHC capture, washing and elution steps, making use of the instrument’s ability to dispense, incubate and push through the necessary buffer solutions. It is important to note that we aimed to develop a workflow that is universally applicable, regardless of the number of MHC molecules present in the samples. By keeping the reagents and conditions in the workflow constant regardless of the input amount, this inherently means that the workflow will perform suboptimal for input amounts far above or below the working range established here. It is possible that the protocol for extreme low input samples could further be optimized, however, one caveat is that these optimizations would be heavily biased toward the sample type used (in this case JY cells) and not extrapolate to other types of input material. As an alternative approach for low-input immunopeptidomics, microfluidics set-ups have emerged that further miniaturize immunopeptidomics sample preparation with minimal sample transfers. However, these setups require highly specialized equipment and expertise for implementation while only processing a single sample each time, making this approach impractical for projects demanding higher throughput.

For maximal performance, low-input sample preparation should be coupled to sensitive MS data acquisition. To this end, we analyzed our samples on a timsTOF SCP instrument operating in DDA-PASEF mode, leveraging ion mobility-*m/z* peptide precursor selection polygons tailored for immunopeptidomics (28, 32, 33) (Supplemental Fig. S1). Importantly, the cell input numbers of the ultrasensitive series (20,000–640,000 cells) reported reflect the actual numbers used for lysis, more closely mimicking real-life applications compared to the cell equivalents that are sometimes used for method optimization (28, 32). In these experiments, DDM did not increase the number of identified immunopeptides, indicating no benefit of this surfactant for low-input immunopeptidomics in our setup. However, it is important to note that immunopeptide identification numbers are largely dependent on the used cell line and its MHC expression levels, making comparisons across studies and cell types difficult. To further maximize reliable immunopeptide identification, we applied advanced data analysis by integrating the results of four independent search engines that were rescored by TIMS^2^Rescore (52), as recently described (33). In future work, we plan to couple our platform to alternative MS acquisition methods, including data-independent acquisition-PASEF (30, 36, 75), or to other acquisition schemes such as Slice-PASEF (76) or Synchro-PASEF (77). Alternatively, the high sensitivity and speed offered by Orbitrap Astral instruments holds great promise for low-input immunopeptidomics (29). In terms of sample preparation, we envision to improve the sensitivity of our platform even further by using magnetic beads (42, 46) along with biotinylated MHC antibodies (35, 45) for IP, to work with even smaller sample volumes and to prevent endogenous antibody binding to protein-A or -G beads when processing tissue samples. The latter could also be achieved by introducing a standard preclearing step, which, together with more stringent wash steps, might further reduce the observed background of non-MHC binding peptides in our experiments. Interestingly, MAE has regained interest and was shown to offer specific and sensitive enrichment of MHC class I peptides as a cost-effective alternative for antibody-based IP (29, 30, 31). However, due to its need for live cells in suspension, MAE has limited applicability to solid tissue samples and frozen cells (31, 78), though it was successfully applied to clinical tissue fragments (29). In addition, so far it has only been reported for MHC-I, limiting its applicability to samples where the immunopeptidome of both MHC classes are of interest.

In this study, we have also demonstrated the ability of our immunopeptidomics platform to identify bacterial antigens in a lower-input setting using 16 million infected macrophages. This input downscaling substantially decreases the upfront cell culturing and hands-on time, which makes the procedure compatible with higher biosafety level pathogens for which the maximal number of cells that can be infected is limited. The lower input also allows quantitative immunopeptidomics analysis on multiple replicates and conditions. Firstly, we performed antigen discovery for *L. monocytogenes*, a foodborne model pathogen for which we previously gained a comprehensive view on its presented antigens, allowing us to cross-reference the *Listeria* antigens identified by the low-input platform. Despite a 30-fold reduction in input amount, we identified 50 MHC-I peptides from 35 *Listeria* proteins in infected U937 macrophages. Of these 35 antigens, 12 were identified in previous *Listeria* immunopeptidomics studies with high cell number inputs (8, 33, 36), including previously detected immunodominant antigens and virulence factors such as LLO, OppA, inlC, and inlB. After this verification, we applied our platform on U937 cells infected with BCG, an attenuated *M. bovis* strain. This analysis uncovered 41 MHC-I peptides from 32 BCG proteins, of which groEL2, groES, hupB and embC were also detected by previous BCG antigen discovery studies in higher input analyses (9, 10, 68), while IniB was picked up in *M. tuberculosis*-infected U937/A2 cells (69). Aside from the identification of pathogen-derived immunopeptides, bacterial infection can remodel the host immunopeptidome (14, 15). We previously reported how *Listeria* infection promoted presentation of immune-related self proteins in spleens of infected mice (15). Similarly, we here report how BCG infection of U937 macrophages leads to increased MHC-I presentation of host proteins partaking in immune pathways related to immune signaling and phagocytosis (Fig. 4), pathways that were previously reported to be upregulated in BCG-infected macrophages (79). From a technical perspective, these analyses demonstrated the excellent quantitative reproducibility of our immunopeptidomics platform, with an average Pearson correlation of 0.88 between immunopeptide intensities in uninfected biological replicate samples. Together, these data validate our platform for biological immunopeptidomics applications where a combination of high sensitivity, high throughput, and quantitative performance are required.

## Data Availability

The MS proteomics data have been deposited to the ProteomeXchange Consortium (http://proteomecentral.proteomexchange.org) *via* the PRIDE partner repository (80) with the dataset identifier PXD062352. Annotated MS/MS spectra of JY cell input experiments and bacterial infection experiments and used protein databases are accessible at the Zenodo repository under https://doi.org/10.5281/zenodo.19368946.

## Supplemental data

This article contains supplemental data (8, 26, 28, 40, 41, 42, 43, 44, 45, 46, 51, 52, 53, 54, 55).

## Conflict of Interest

The authors declare no competing interests.
