# Supplementary material for: A Platform for High-throughput and Ultrasensitive Immunopeptidomics
Source: Mol Cell Proteomics. 2026 May 18;25(6):101590. doi: 10.1016/j.mcpro.2026.101590 (PMC13284484; doi:10.1016/j.mcpro.2026.101590)
Supplement: Supplemental Data [file mmc11.pdf]

Supplemental Data

A platform for high-throughput and ultrasensitive immunopeptidomics

Adillah Gul[1,2,*], Laura Van Moortel[1,2,*], Patrick Willems[1,2,*], Ilke Aernout[3,7], Laura Pedró-Cos[4], Kia C. Ferrell[4], Katie Boucher[1,2,5], An Staes[1,2,5], Simon Devos[1,2,5], Ine Lentacker[3,7], Bart Vandekerckhove[6,7,8], Caroline Demangel[4], Fabien Thery[1,2,#], Francis Impens[1,2,#]

Author affiliations:

[1] VIB Center for Medical Biotechnology, VIB, Ghent, Belgium

[2] Department of Biomolecular Medicine, Ghent University, Ghent, Belgium

[3] Ghent Research Group on Nanomedicines, Ghent University, Ghent, Belgium

[4] Institut Pasteur, Université Paris Cité, Inserm U1224, Immunobiology and Therapy Unit, Paris, France

[5] VIB Proteomics Core, VIB, Ghent, Belgium

[6] Department of Diagnostic Sciences, Ghent University, Ghent, Belgium.

[7] Cancer Research Institute Ghent (CRIG), Ghent, Belgium.

[8] GMP Unit cell Therapy, Ghent University Hospital, Ghent, Belgium.

TABLE OF CONTENTS

Supplemental Table S1	Set collision energy scheme for timsTOF SCP acquisition of MHC-I and MHC-II peptides.
Supplemental Table S2	Overview of plate- and microfluidics-based immunopeptidomics approaches using immunopurification.
Supplemental Figure S1	TimsTOF SCP polygons used for MHC-I and MHC-II peptide acquisition.
Supplemental Figure S2	Effect of lysate volume and concentration on IP efficiency.
Supplemental Figure S3	Optimization of MHC-I and MHC-II IP methods.
Supplemental Figure S4	Optimization of the IP elution conditions.
Supplemental Figure S5	Optimization of the peptide purification method.
Supplemental Figure S6	Identified peptide length histograms of MHC-II pulldowns testing different antibodies and without prior MHC-I pulldown.
Supplemental Figure S7	Identification boosting by multi-engine rescoring per JY cell input.
Supplemental Figure S8	Reproducibility of MHC-I peptide identification and quantification.
Supplemental Figure S9	Reproducibility of MHC-II peptide identification and quantification.
Supplemental Figure S10	JY MHC class I and II predicted binding strengths.
Supplemental Figure S11	Immunopeptidomics on HeLa and U937 cell inputs.
Supplemental Figure S12	Peptide length and Gibbs clusters of ultrasensitive JY cell inputs.
Supplemental Figure S13	Identification boosting by multi-engine rescoring per JY cell dilution (ultrasensitive series).
Supplemental Figure S14	Ultrasensitive immunopeptidomics in presence of n-dodecyl-β-D-maltoside.

Supplemental Figure S15	Predicted binding strength for identified immunopeptides in the ultrasensitive JY cell input series.
Supplemental Figure S16	Non-binders in low cell amounts are enriched in protein contaminants originating from sample preparation.
Supplemental Figure S17	Immunopeptidomics quality control of U937 cell cultures infected by *Listeria monocytogenes* and *Mycobacterium bovis* BCG.
Supplemental Figure S18	Quantitative reproducibility and variation between BCG-infected and uninfected samples.
Supplemental Methods	Detailed step-by-step immunopeptidomics protocol.
Supplemental Data S1	Identified MHC-I immunopeptide sequences in JY serial cell inputs [XLSX].
Supplemental Data S2	Identified MHC-II immunopeptide sequences in JY serial cell inputs [XLSX].
Supplemental Data S3	Identified MHC-I immunopeptide sequences in HeLa serial cell inputs [XLSX].
Supplemental Data S4	Identified MHC-I immunopeptide sequences in U937 serial cell inputs [XLSX].
Supplemental Data S5	Identified MHC-I immunopeptide sequences in JY low input serial cell inputs without the nonionic surfactant n-dodecyl-β-D-maltoside (DDM) [XLSX].
Supplemental Data S6	Identified MHC-I immunopeptide sequences in JY low input serial cell inputs with the nonionic surfactant n-dodecyl-β-D-maltoside (DDM) [XLSX].
Supplemental Data S7	FragPipe HLA peptidome quantitative analysis of the JY low input serial inputs without the nonionic surfactant n-dodecyl-β-D-maltoside (DDM) [XLSX].
Supplemental Data S8	Identified human self-peptides and *Listeria* peptides during infection in U937 cells [XLSX].
Supplemental Data S9	Identified human self-peptides and BCG peptides during infection in U937 cells [XLSX].
Supplemental Data S10	Differential peptide abundance analysis during BCG infection [XLSX].

Supplemental Tables

Supplemental Table S1. Set collision energy scheme for timsTOF SCP acquisition of MHC-I and MHC-II peptides.

$1/K_0$ [Vs cm^{-2}]	Collision energy [eV]
0.7	20
1.06	30
1.16	40
1.34	40
1.68	70

Supplemental Table S2. Overview of plate- and microfluidics-based immunopeptidomics approaches using immunopurification. Details on sample preparation, data acquisition and final peptides identified (1% FDR) are provided. Missing information is labeled as not specified (n.s.). Abbreviations: LAUD, lung adenocarcinoma; MHC-Ip, MHC class I peptides; MHC-IIp, MHC class II peptides; PDAC, pancreatic ductal adenocarcinoma; Prot-A, protein A.

Study	Input tissue (g) or cells (number)	Sample preparation (lysis, IP, elution and purification)						Data acquisition and analysis	
		Antibody to bead ratio	Lysis volume	Automatization (# samples)	IP condition	MHCp elution	MHCp purification	MS instrument	Number of peptides identified (FDR 1%)
Chong et al. (26)	Meningioma tissue (1g) and 7 cell lines (100 MY)	5 mg/mL Prot-A Sepharose	10 mL (tissue), 1 mL (cells)	96 well microplate 3 µm glass fiber and 10 µm polypropylene membranes (n=96)	Gravity flowthrough at 4°C	1% TFA	Sep-Pak tC18 100 mg	QExactive HF	MHC-Ip: 3,293-13,696 MHC-IIp: 7,210-10,060
Zhang et al. (40)	Raji cells (100 MY)	250 µg/mL Prot-A cartridges (AssayMAP Bravo)	0.8 mL	96 well deep plate for lysate (n=96)	Flow rate 10 µL/min (80 min)	10% acetic acid	10 kDa spin column, AssayMAP's RP-S cartridge	LTQ Orbitrap Elite, Fusion Lumos	MHC-Ip: 5,578 MHC-IIp: 8,250
Pollock et al. (41)	MC38 and GRANTA cells (250 MY)	1mg/mL Prot-A cartridge	5 mL	96-well deep plate for lysate (n=96)	Flow rate 20 µL/min	1% acetate	C18 Cartridges	Fusion Lumos Tribrid	MHC-Ip: > 4,000
Abelin et al. (44)	LAUD tumor (50 mg)	0.015 mg/37.5µL Gammabind Sepharose	1.2 mL	IP in tubes and transferred to 10 µm PE fritted plate (n.s.)	Incubating tubes with beads and lysate on a rotor at 4 °C (3 h)	10% acetic acid	Sep-Pak tC18 40 mg	Orbitrap Exploris 480	MHC-Ip: 8,278-13,727 MHC-IIp: 1,123-9,726
Lim Kam Sian et al. (42)	MDA-MB-231 cells (0.5 to 50 MY)	0.5 mg/80 µL MagReSyn Prot-A	300-600 µL	Kingfisher 96-well plate (n=12)	1 h	0.1% TFA	C-18 stage tips	Orbitrap Exploris 480	MHC-Ip: ~ 400-3,000
Phulphagar et al. (28)	Melanoma tumor (25 mg) and A375 and PDAC cell lines (1 to 40 MY)	0.015 mg/37.5 µL Gammabind Sepharose	1.2 mL	IP in 96-well deep plate and transferred to 10 µm PE fritted plate (n=96)	End-to-end rotation (3h)	10% acetic acid	Sep-Pak tC18 40 mg	TimsTOF SCP	MHC-Ip: 7,000-15,000 MHC-IIp: up to 15,000 from 40 MY
Feola et al. (45)	JY cells (1 to 50 MY), ovarian tumor (10 to 60 mg), organoids	45 µg biotinylated antibody	n.s.	IP on PeptiCHIP (n=1)	n.s.	7% acetic acid in 50% meOH	SepPac-C18 cartridges	TimsTOF Pro	MHC-Ip: 1,800-5,590 from 1 to 50 MY JY cells
Li et al. (43)	Melanoma tumor (5 to 50 mg), RA957 cells (0.2 to 10 MY)	3 mg/mL Prot-Sepharose	100-200 µL	IP on Chip (n=12)	At lowest flow rate	10% acetic acid	C-18 cartridges	Q Exactive HF-X	MHC-Ip: 4,000-15,000
Tanuwidjaya et al. (46)	500 µL plasma	100 µg/100 µL MagReSyn Prot-A	n.s.	Kingfisher, 96-well plate (n=96)	1 h	10% acetic acid	SDB-XC stage tips	Orbitrap Exploris 480	MHC-Ip: 1,257-4,226 from 100 µL to 1 mL plasma

Supplemental Figures

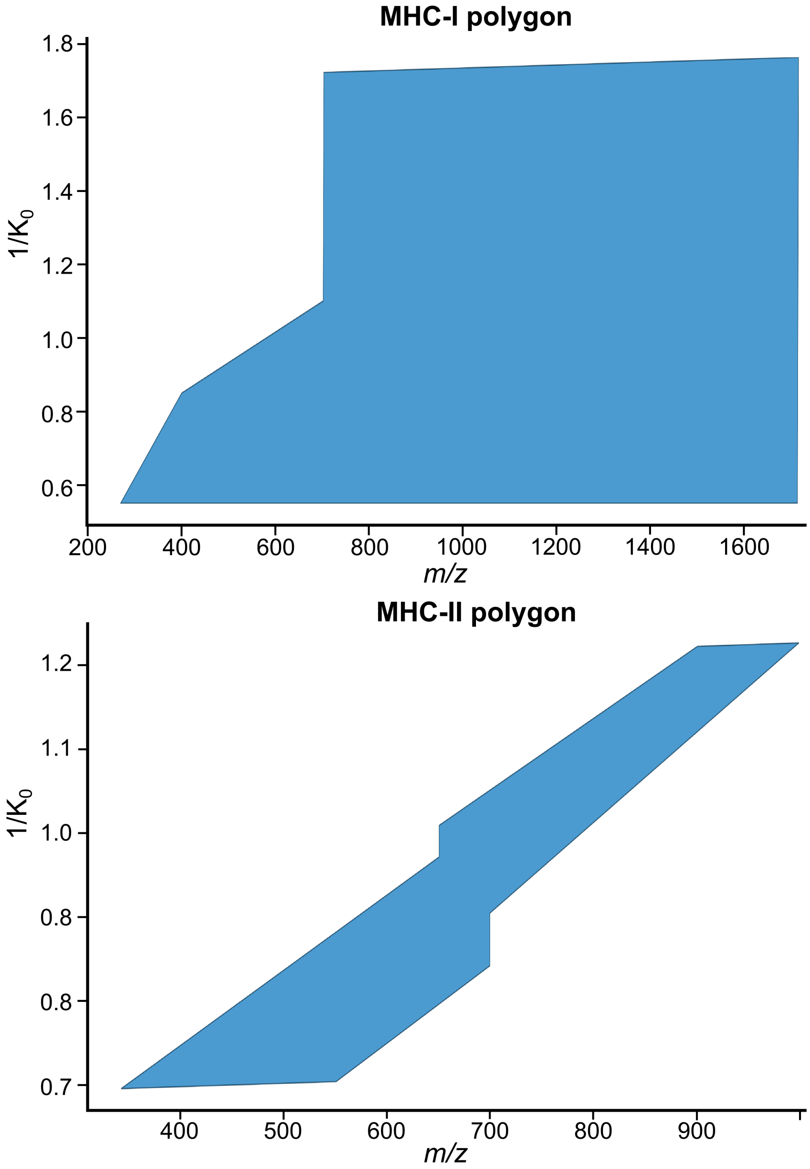

Supplemental Figure S1. **TimsTOF SCP polygons used for MHC-I and MHC-II peptide acquisition.** The blue area indicates the polygon across the inverse reduced ion mobility ($1/K_0$) vs *m/z* dimension used for MHC-I (*Top*) and MHC-II peptides (*Bottom*). The MHC-I polygon has an extended upper ion mobility range to allow inclusion of singly charged peptides. Polygon figures were extracted from method reports generated by timsCompare (v1.2) (https://github.com/kronigert/timsCompare).

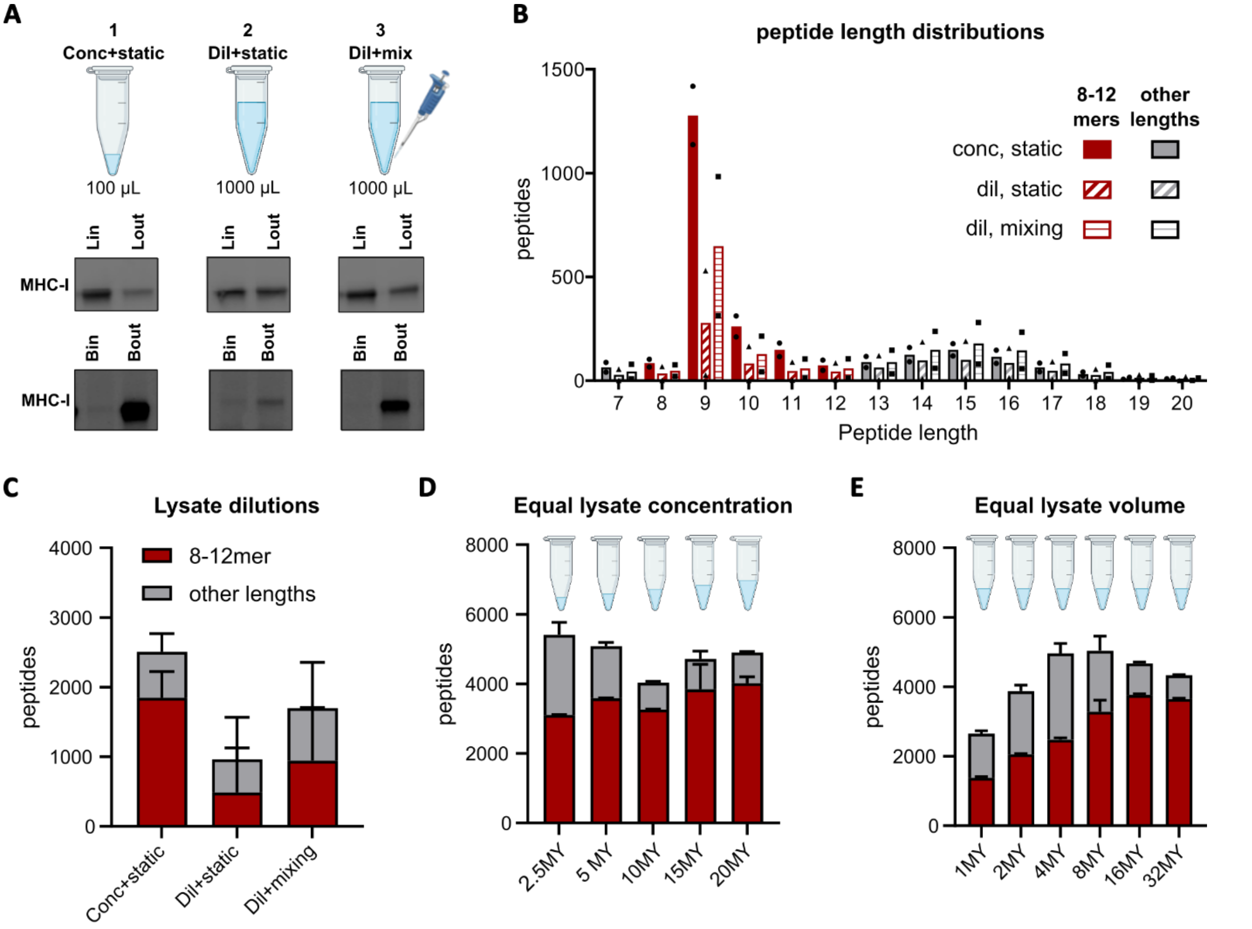

Supplemental Figure S2. **Effect of lysate volume and concentration on IP efficiency.** All data are representative of two technical replicates per condition. Washing and peptide clean-up steps were performed according to a standard immunopeptidomics workflow (8). Samples were analyzed on a timsTOF SCP instrument coupled in-line to an EvoSep One chromatography system (25% sample injection). Peptides were identified using PEAKS (v11.0) (51). **(A-C)** 10 million (MY) JY cells were lysed in 100 µL ("Conc") or 1000 µL ("Dil") lysis buffer and incubated for 1 h at 4°C with 300 µg W6/32 HLA class I antibody crosslinked to 100 µL packed protein A beads in 96-well plates, with or without mixing. **(A)** Control immunoblots incubated with antibody against HLA class I. Abbreviations: Lin, lysate before IP; Lout, lysate after IP; Bin, beads before IP; Bout, beads after IP. **(B)** Identified peptide length distribution for all three conditions. Data points indicate technical replicates. **(C)** Number of identified 8-12mer peptides (red) and peptides of other lengths in all three conditions. **(D)** 2.5 to 20 MY JY cells were lysed in 100 µL lysis buffer per 10 MY cells and incubated for 1 h at 4°C with 300 µg W6/32 antibody crosslinked to 100 µL packed protein A beads. The graph represents the number of identified 8-12mer peptides (red) and peptides of other lengths identified per condition. **(E)** 1 to 32 MY JY cells were lysed in 100 µL lysis buffer and incubated for 1 h at 4°C with 300 µg W6/32 antibody crosslinked to 100 µL packed protein A beads. The graph represents the number of identified 8-12mer peptides (red) and peptides of other lengths identified per condition. Figure created with BioRender.com.

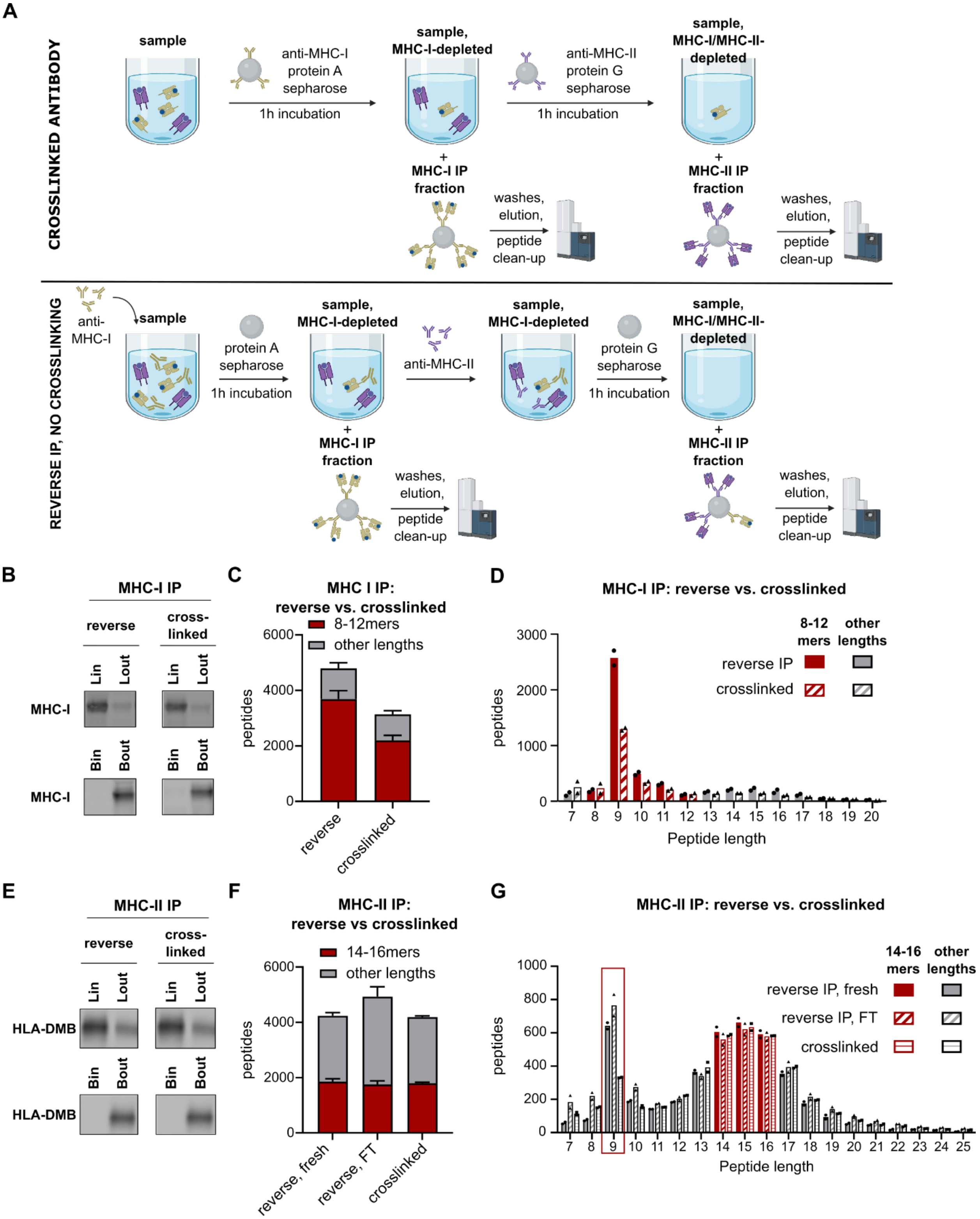

Supplemental Figure S3. Optimization of MHC-I and MHC-II IP methods. 10 million JY cells were lysed in 100 µL lysis buffer and subjected to various IP methods, followed by washing steps and peptide clean-up according to the standard immunopeptidomics workflow (8). Samples were run on a timsTOF SCP instrument coupled in-line to an Evosep One chromatography system (25% sample injection). Data

represent the average of two technical replicates. Peptides were identified using PEAKS (v11.0) (51). **(A)** Workflow overview for reverse IP and IP with crosslinked antibody. Figure created with Biorender. **(B-D)** Comparison of reverse IP and IP with crosslinked antibody for MHC class I. In the reverse IP, 100 µL lysate was mixed with 300 µg W6/32 antibody diluted in 100 µL of 50 mM Tris-HCl, 150 mM NaCl (pH 7.5) before 1 h incubation with 100 µL packed protein A beads (4°C). In the regular IP, 100 µL lysate was mixed with 100 µL of 50 mM Tris-HCl, 150 mM NaCl (pH 7.5) and incubated with 300 µg W6/32 antibody crosslinked to 100 µL packed protein A beads for 1 h at 4°C. **(B)** Control immunoblots, incubated with antibody against HLA class I. Lin, lysate before IP; Lout, lysate after IP; Bin, beads before IP; Bout, beads after IP. **(C)** Number of identified 8- to 12mer peptides (red) and peptides of other lengths (grey) in both conditions. **(D)** Peptide length distributions for both conditions. **(E-G)** Comparison of reverse IP and IP with crosslinked antibody for MHC class II. In the reverse IP, 175 µL fresh lysate ('reverse, fresh') or 175 µL lysate flow-through after MHC-I IP ('reverse, FT') was mixed with 150 µg PdV5.2 MHC class II antibody diluted in 125 µL of 50 mM Tris-HCl, 150 mM NaCl (pH 7.5) before 1 h incubation with 100 µL packed protein G beads (4°C). In the regular IP, 100 µL lysate was mixed with 100 µL of 50 mM Tris-HCl, 150 mM NaCl (pH 7.5) and incubated with 150 µg PdV5.2 MHC class II antibody crosslinked to 100 µL packed protein G beads for 1h at 4°C. **(E)** Control immunoblots, incubated with antibody against HLA-DMB. Lin, lysate before IP; Lout, lysate after IP; Bin, beads before IP; Bout, beads after IP. **(F)** Number of identified 14- to 16-mer peptides (red) and peptides of other lengths (grey) identified in all conditions. **(G)** Peptide length distributions for all conditions.

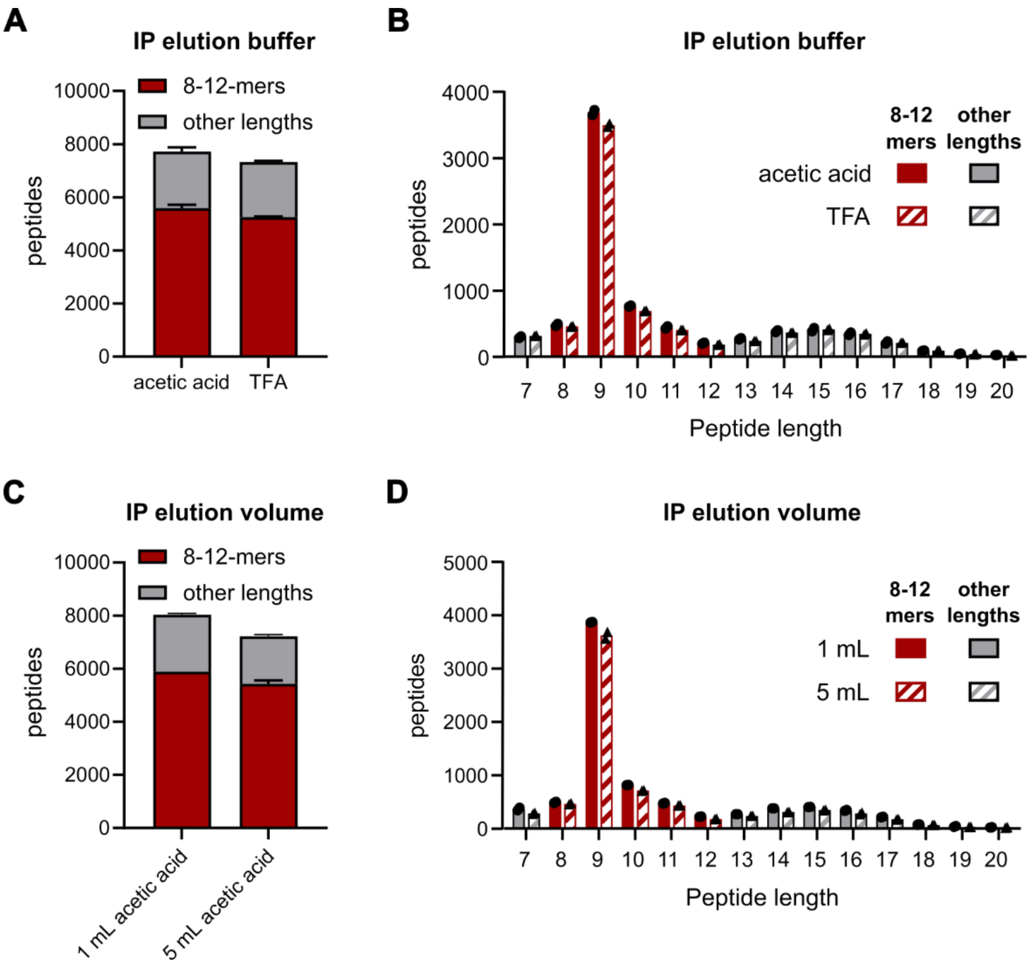

Supplemental Figure S4. Optimization of the IP elution conditions. Ten million JY cells were lysed in 100 µL lysis buffer and subjected to MHC-I IP using 300 µg W6/32 antibody crosslinked to 100 µl packed protein A beads. Peptide clean-up was performed according to the standard immunopeptidomics workflow (8), followed by sample injection on a timsTOF SCP instrument with the Evosep One chromatography system (25% sample injection). Data represent the average of two technical replicates. Peptides were identified using PEAKS (v11.0) (51). **(A, B)** Comparison of MHC-I IP elution using 5 mL 10% acetic acid or 5 mL 0.1% trifluoroacetic acid (TFA). **(C, D)** Comparison of MHC-I IP elution using 5 mL or 1 mL 10% acetic acid. **(A, C)** Number of identified 8-12mer peptides (red) and peptides of other lengths (grey) identified in both conditions. **(B, D)** Peptide length distribution for both conditions.

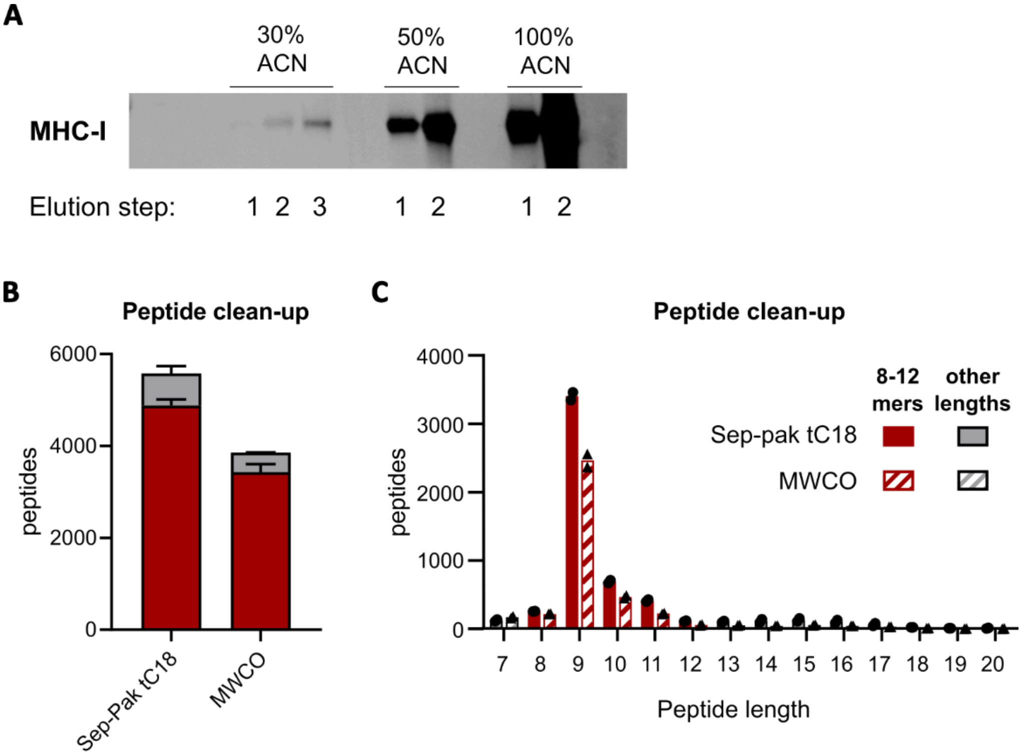

Supplemental Figure S5. **Optimization of the peptide purification method.** Ten million JY cells were lysed in 100 µL lysis buffer and subjected to MHC-I IP according to standard workflow. **(A)** Immunoblot analysis showing the elution of HLA class I proteins from Sep-Pak tC18 resin upon multiple elution steps in 0.1% TFA with increasing percentages of ACN. **(B-C)** Comparison of the number of identified peptides following clean-up using Sep-Pak tC18 96-well plates or 10 kDa molecular weight cut-off (MWCO) filters. 10 million JY cells were lysed in 100 µL lysis buffer and subjected to MHC-I IP using 300 µg W6/32 antibody crosslinked to 100 µL packed protein A beads. Peptides were purified using either Sep-Pak tC18 96-well plates (100 mg) with elution in 25% ACN 0.1% TFA, or using a 10 kDa MWCO filter followed by purification on a Sep-Pak tC18 96-well plate with elution in 40% ACN 0.1% TFA. Samples were run on the timsTOF SCP instrument with the Evosep One chromatography system (25% sample injection). Data represent the average of two technical replicates. Peptides were identified using PEAKS (v11.0) (51). **(B)** Number of identified 8-12mers (red) and peptides of other lengths per condition (grey). **(C)** Peptide length distribution for both conditions.

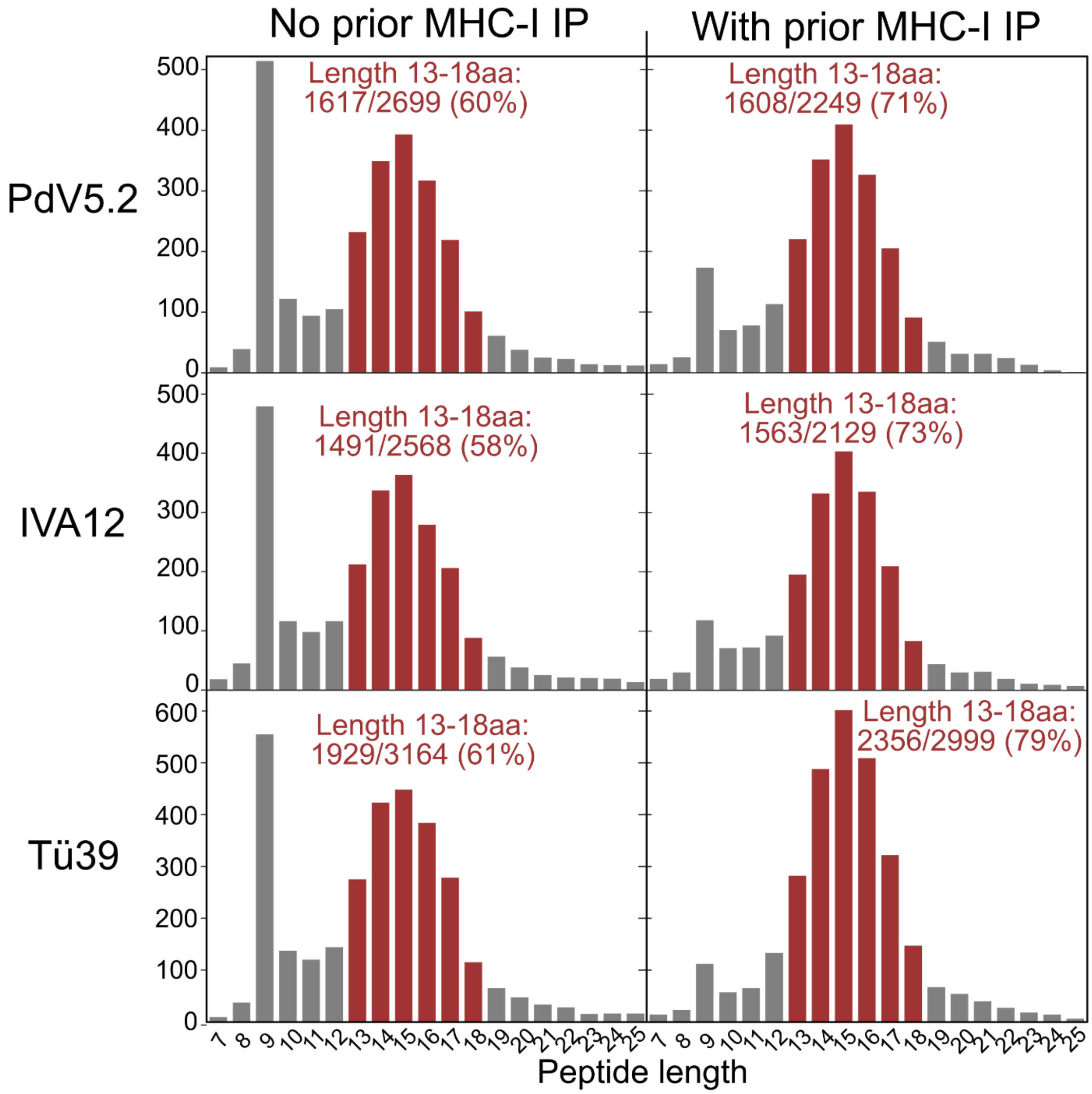

Supplemental Figure S6. Identified peptide length histograms of MHC-II pulldowns testing different antibodies and without prior MHC-I pulldown. Three pan-MHC-II antibodies were tested on the same input of JY cell material: PdV5.2, IVA12 and TU39 (*Top to down*). The number of unique identified peptide sequences was shown in function of peptide length in amino acids. Length 13 to 18 was indicated in red, representing a typical MHC-II compatible length peaking in MHC-II immunopeptidomics. Samples without a prior MHC-I pulldown (*Left*) and with a prior MHC-I pulldown (*Right*) were compared, the latter being a default approach to reduce the aspecific isolation of MHC-I peptides visible here by the 9-mer peak.

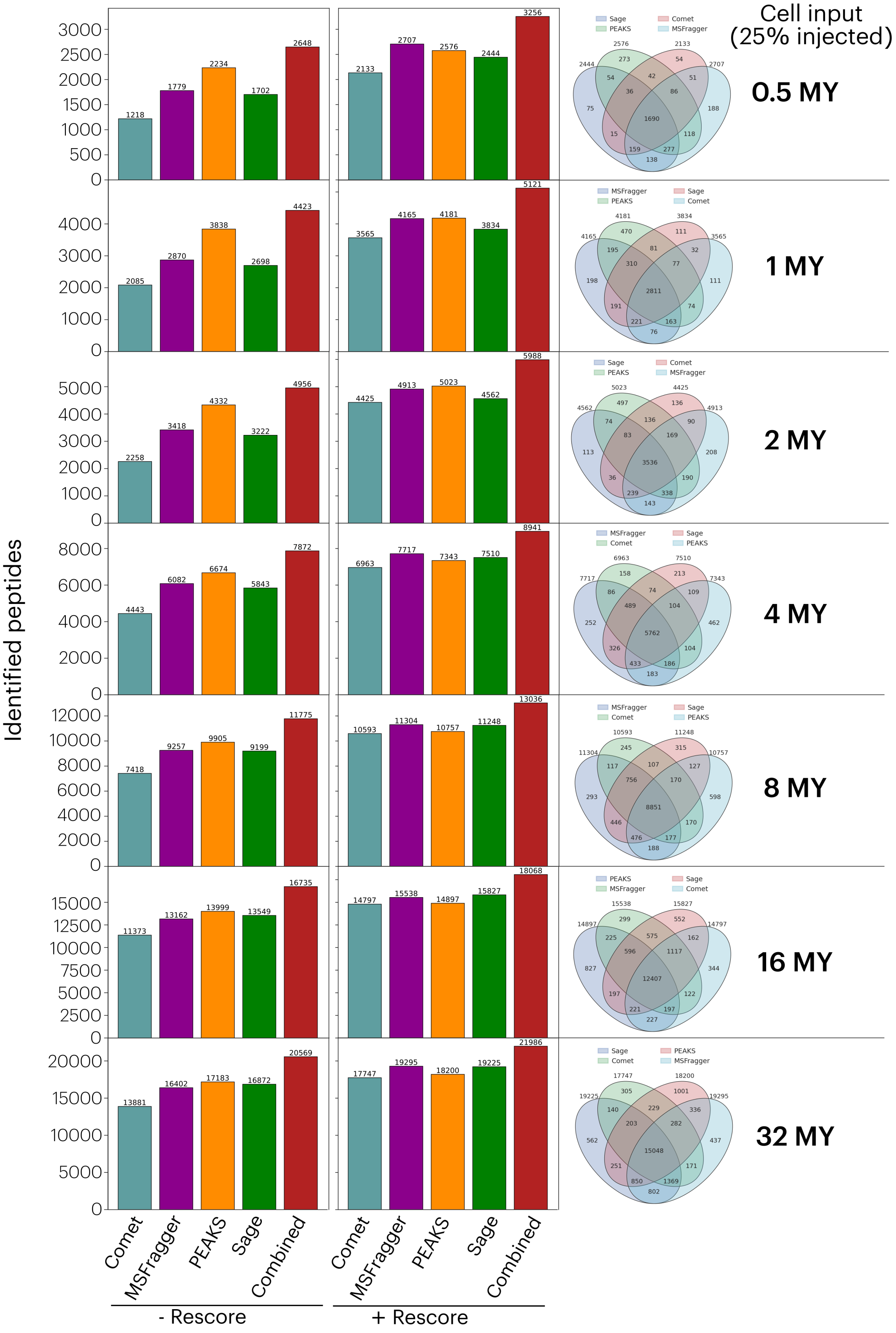

Supplemental Figure S7. **Identification boosting by multi-engine rescoring per JY cell input.** (*Left*) The number of identified peptides per search engine or all four combined was shown prior and after data-driven rescoring by TIMS^{2}Rescore (52). (*Right*) Venn diagram displaying the overlap of peptide sequences identified per search engine (after rescoring).

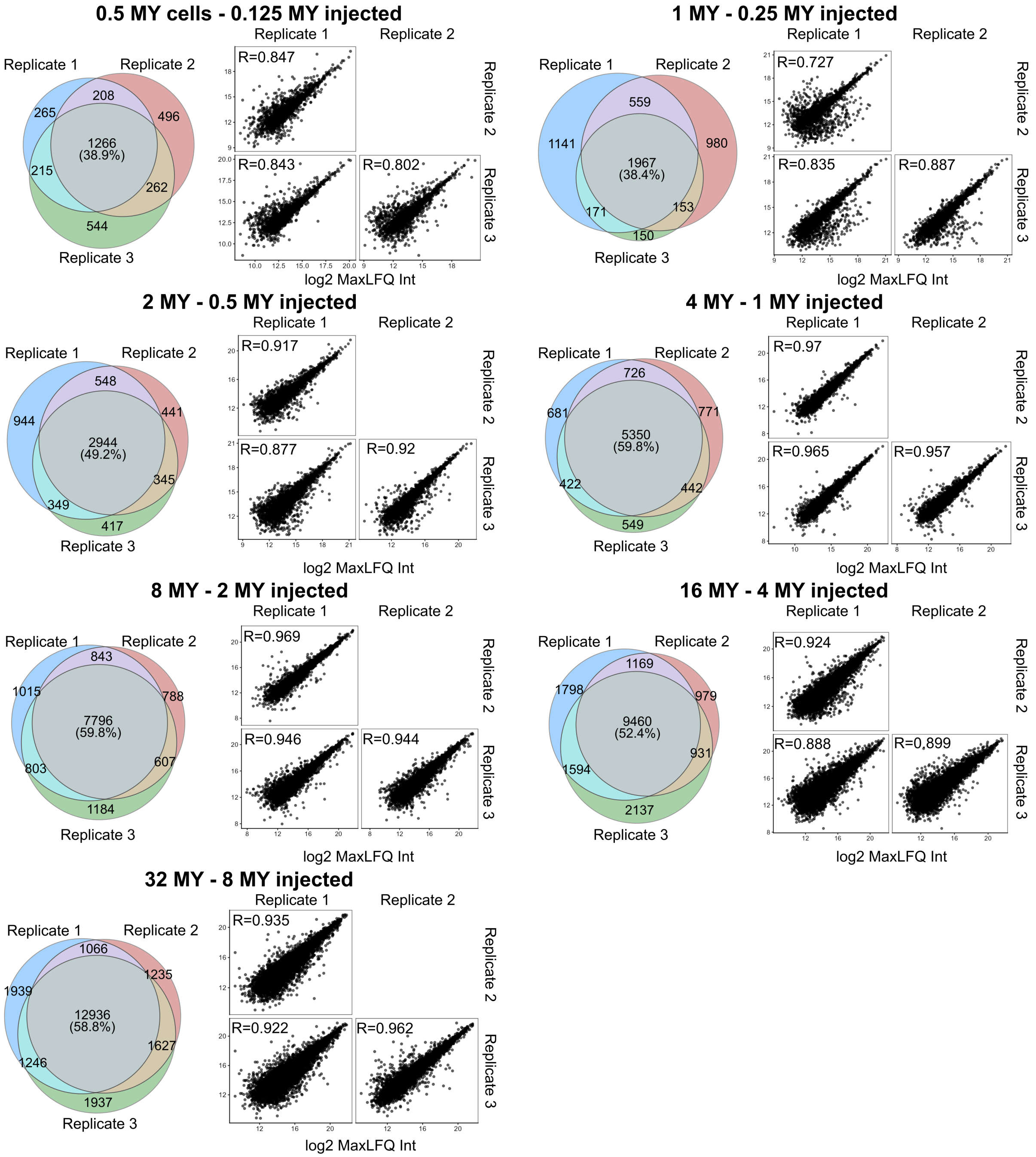

Supplemental Figure S8. **Reproducibility of MHC-I peptide identification and quantification.** For each JY cell input amount (0.5 to 32 million [MY] cells, 25% injected, Data S1), the peptide overlap between replicates is shown in a Venn diagram (*Left*) and pairwise scatter plots of log_2-transformed MaxLFQ peptide intensities outputted by a FragPipe 23.0 HLA-nonspecific search (*Right*). Pearson correlation coefficients are indicated and plots were made using the GGally (doi: 10.32614/CRAN.package.GGally) *ggpairs* function in R.

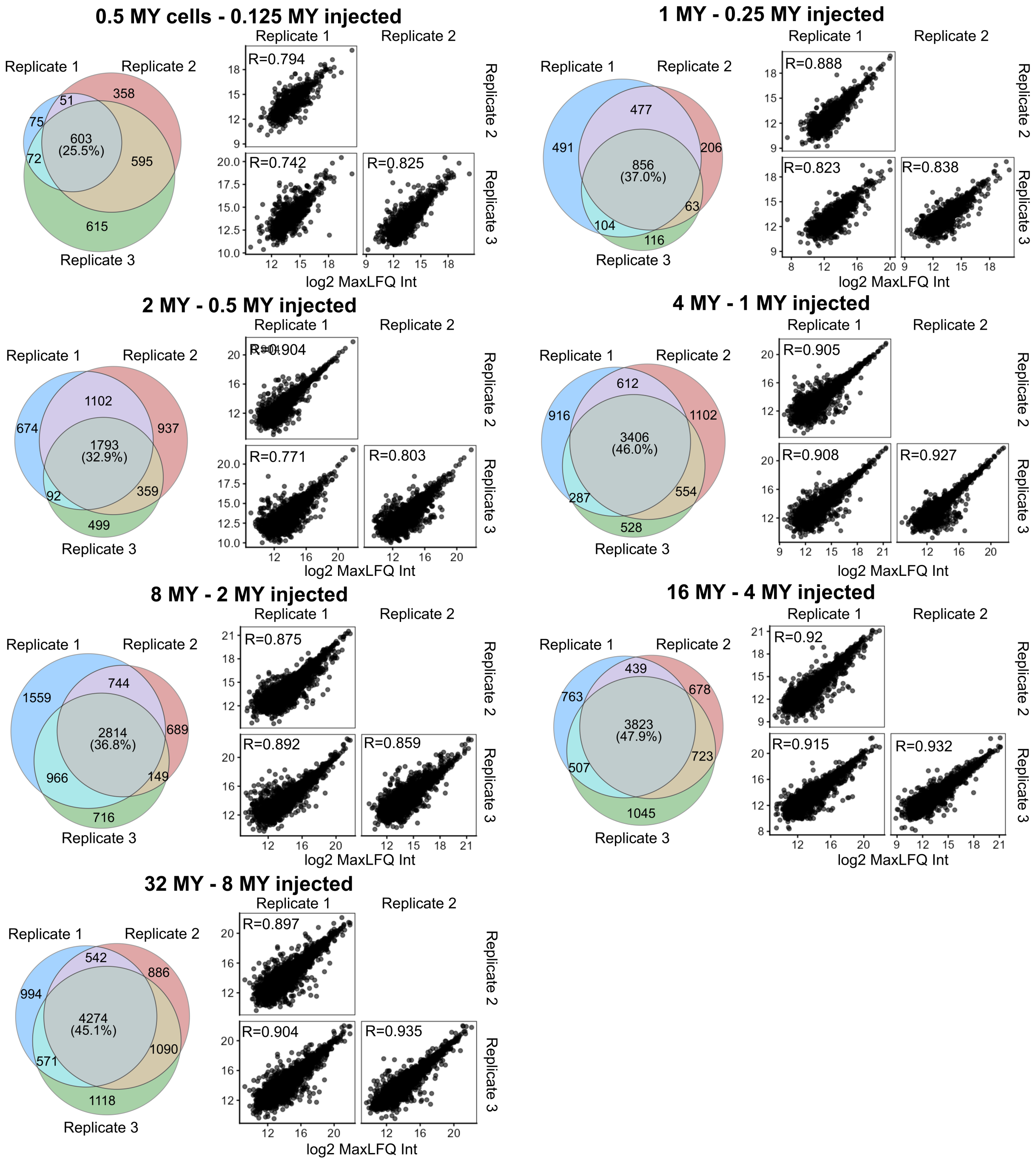

Supplemental Figure S9. **Reproducibility of MHC-II peptide identification and quantification.** For each JY cell input amount (0.5 to 32 million [MY] cells, 25% injected, Data S2), the peptide overlap between replicates is shown in a Venn diagram (*Left*) and pairwise scatter plots of log_2-transformed MaxLFQ peptide intensities outputted by a FragPipe 23.0 HLA-nonspecific search (*Right*). Pearson correlation coefficients are indicated and plots were made using the GGally (doi: 10.32614/CRAN.package.GGally) *ggpairs* function in R.

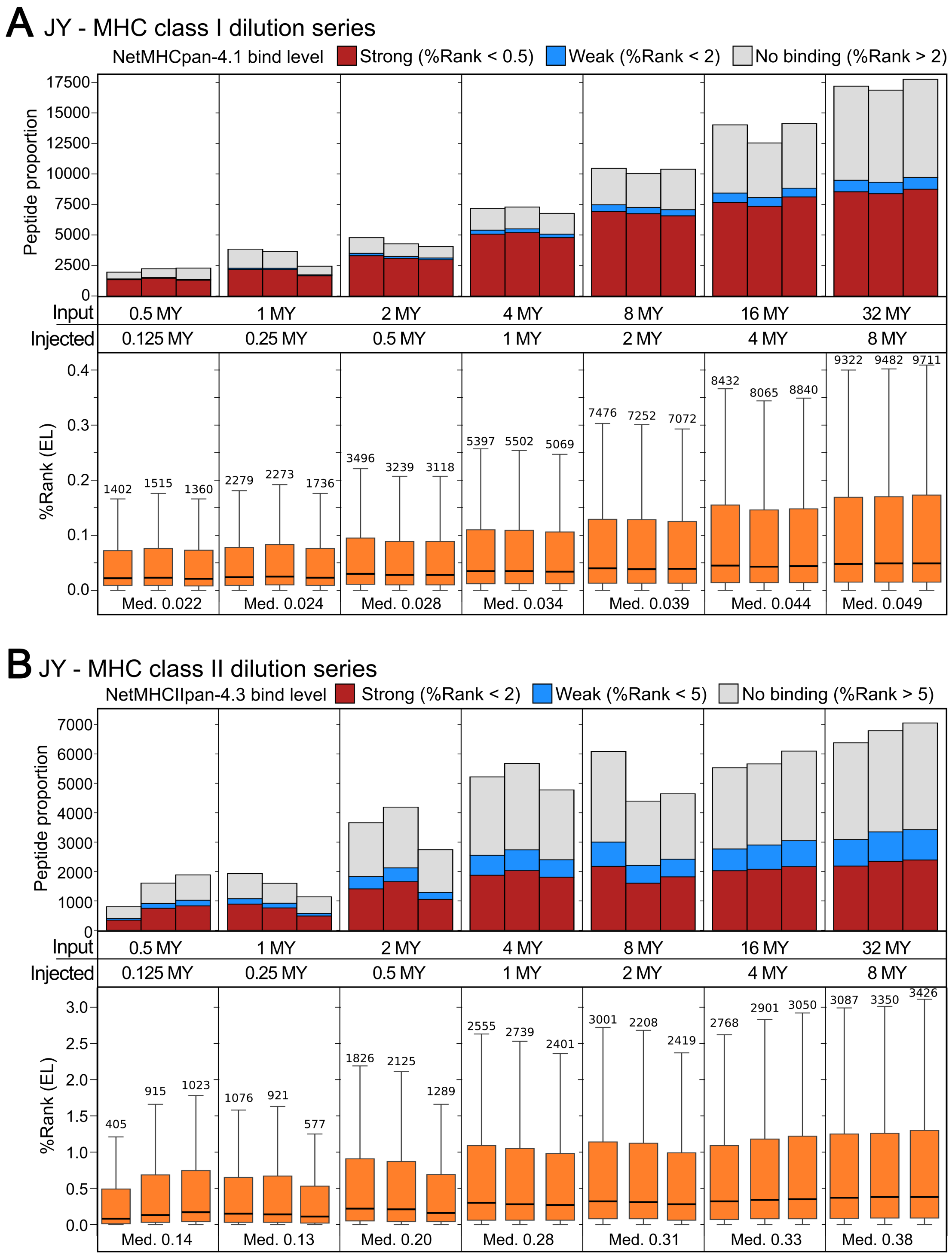

Supplemental Figure S10. JY MHC class I and II predicted binding strengths. (A-B) Identified peptides sequences per sample grouped per JY cell input amount for MHC class I (**A**) and MHC class II (**B**). Each cell input amount was processed and searched independently, and 25% of the input material was injected on timsTOF SCP. (*Top*) Peptides are colored according to predicted MHC binding strength by NetMHCpan-4.1 (53) for MHC class I (**A**) and NetMHCIIpan-4.3 (54) for MHC class II (**B**). (*Bottom*) Boxplot distributions of the %Rank score were shown for predicted binders in each individual sample. Per cell input amount, the median %Rank was displayed for all predicted MHC binders.

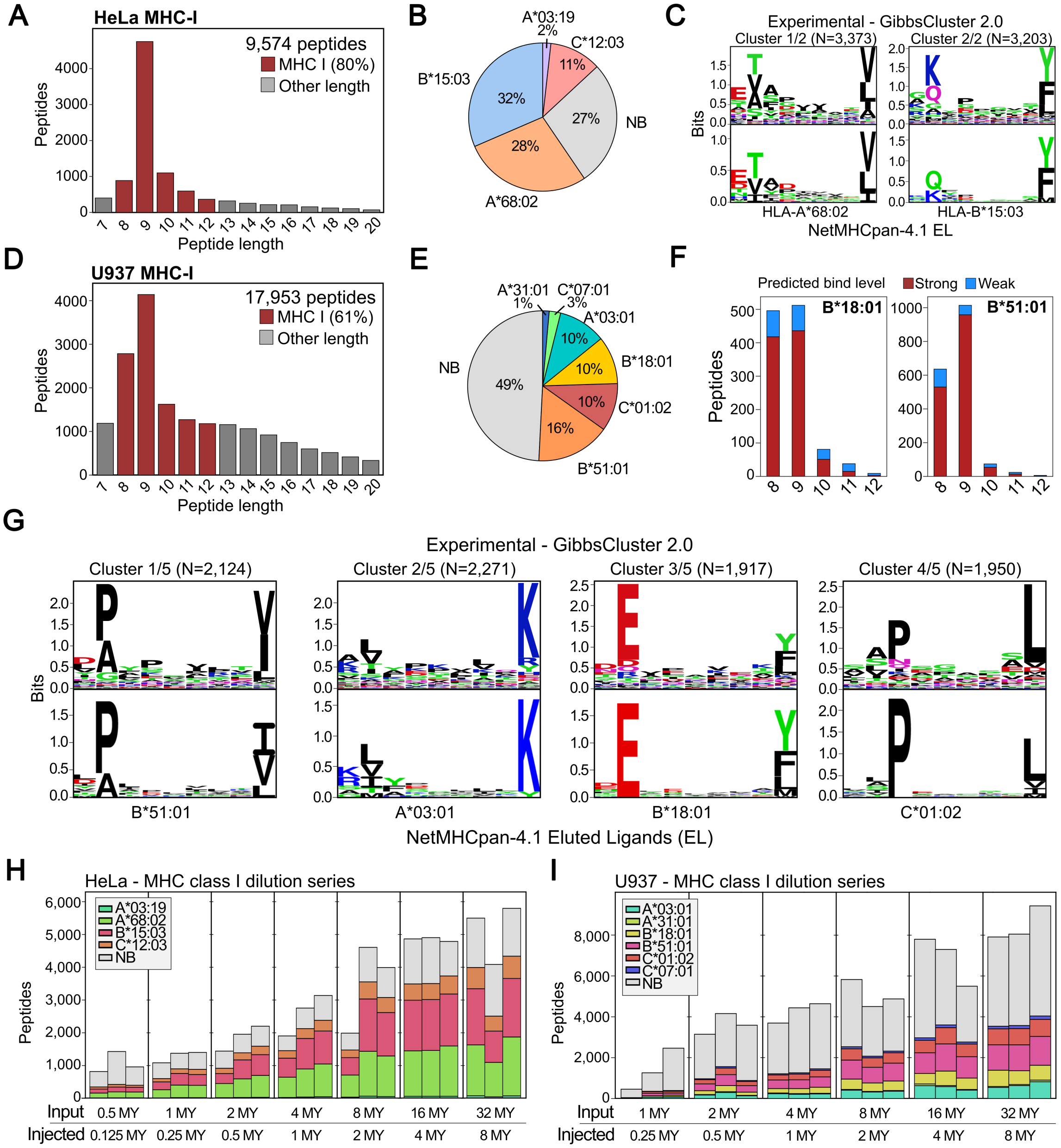

Supplemental Figure S11. Immunopeptidomics on HeLa and U937 cell inputs. (**A, D**) Identified peptide length histogram of MHC class I pulldown in HeLa (**A**) and U937 cells (**D**). (**B, E**) Pie charts indicating the NetMHCpan-4.1 (53) predicted peptide binders to MHC alleles for HeLa (**B**) and U937 cells (**E**). Binders were defined as peptides with a %Rank < 2 and were assigned to the MHC allele with the lowest %Rank. Other peptides were classified as nonbinders (NB, grey). (**C, G**) Unsupervised Gibbs clustering (55) of HeLa (**C**) and U937 (**G**) 8-12mer peptides reveals sequence logos matching MHC class I allele eluted ligand (EL) motifs of netMHCpan-4.1 (53). (**F**) Number of predicted weak and strong peptide binders (%Rank < 2 and < 0.5, respectively) per peptide length to HLA-B*18:01 and HLA-B*51:01 in the U937 dataset. (**H-I**) Identified peptide sequences per sample grouped per cell input amount for HeLa (**H**) and U937 (**I**) cells. Each sample was generated and processed independently, and 25% of the input material was injected for LC-MS/MS analysis on a timsTOF SCP. In case of the HeLa cell series (**H**), the numbers of identified immunopeptides correlated with the measured protein concentration values prior to pulldown, reflecting variation between the biological replicate samples. Peptides are colored according to MHC binding prediction by netMHCpan-4.1 (53).

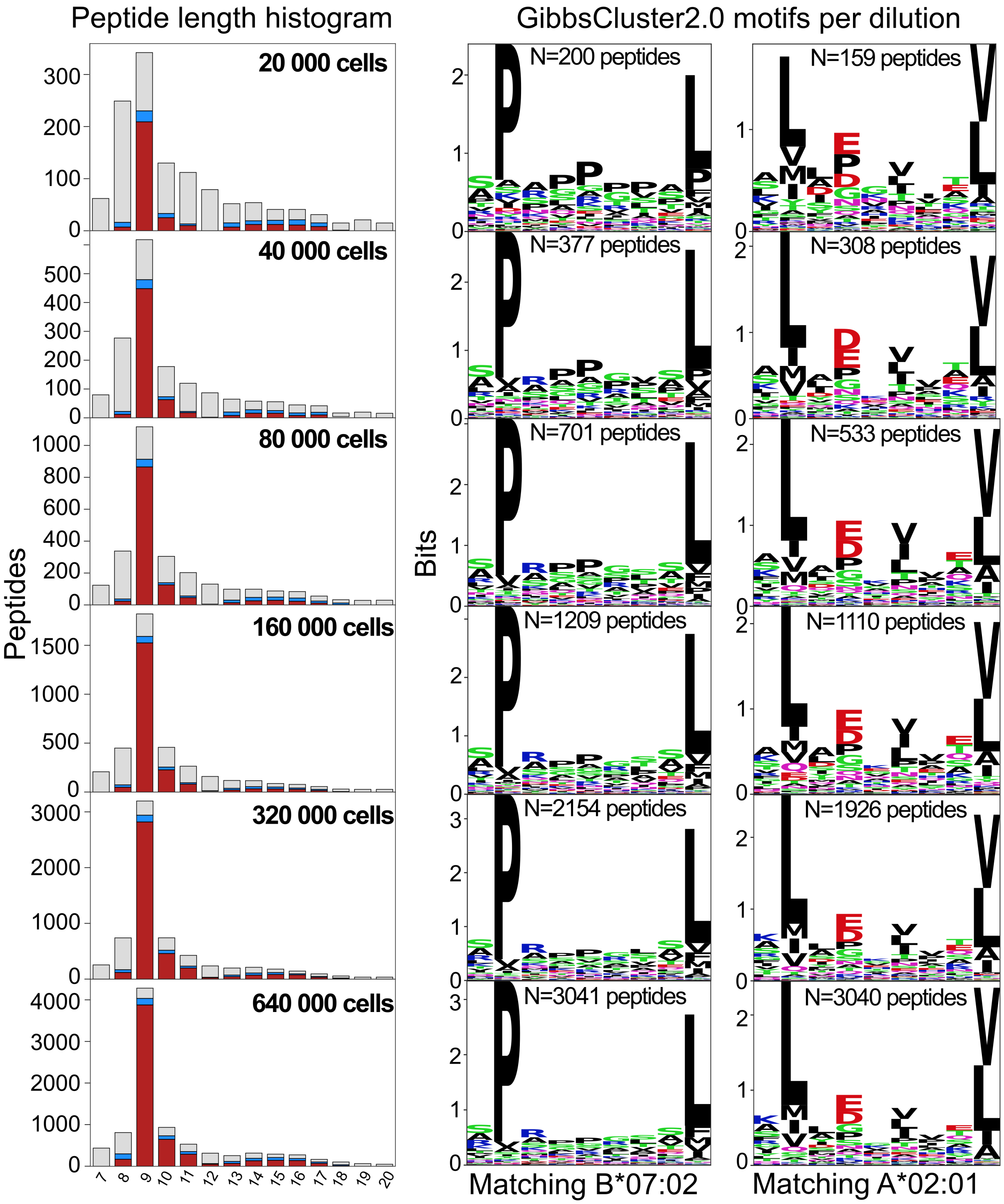

Supplemental Figure S12. Peptide length and Gibbs clusters of ultrasensitive JY cell inputs. Peptide length distributions and sequence motifs corresponding to JY alleles HLA-A*02:01 and HLA-B*07:02 after unsupervised clustering by GibbsCluster2.0 (55) of the identified 8-12mers per JY cell input (from 640,000 to 20,000 cells, *Bottom to top*). In the peptide length histograms, predicted weak and strong peptides binders (%Rank < 2 and < 0.5, respectively) were indicated in blue and red, respectively.

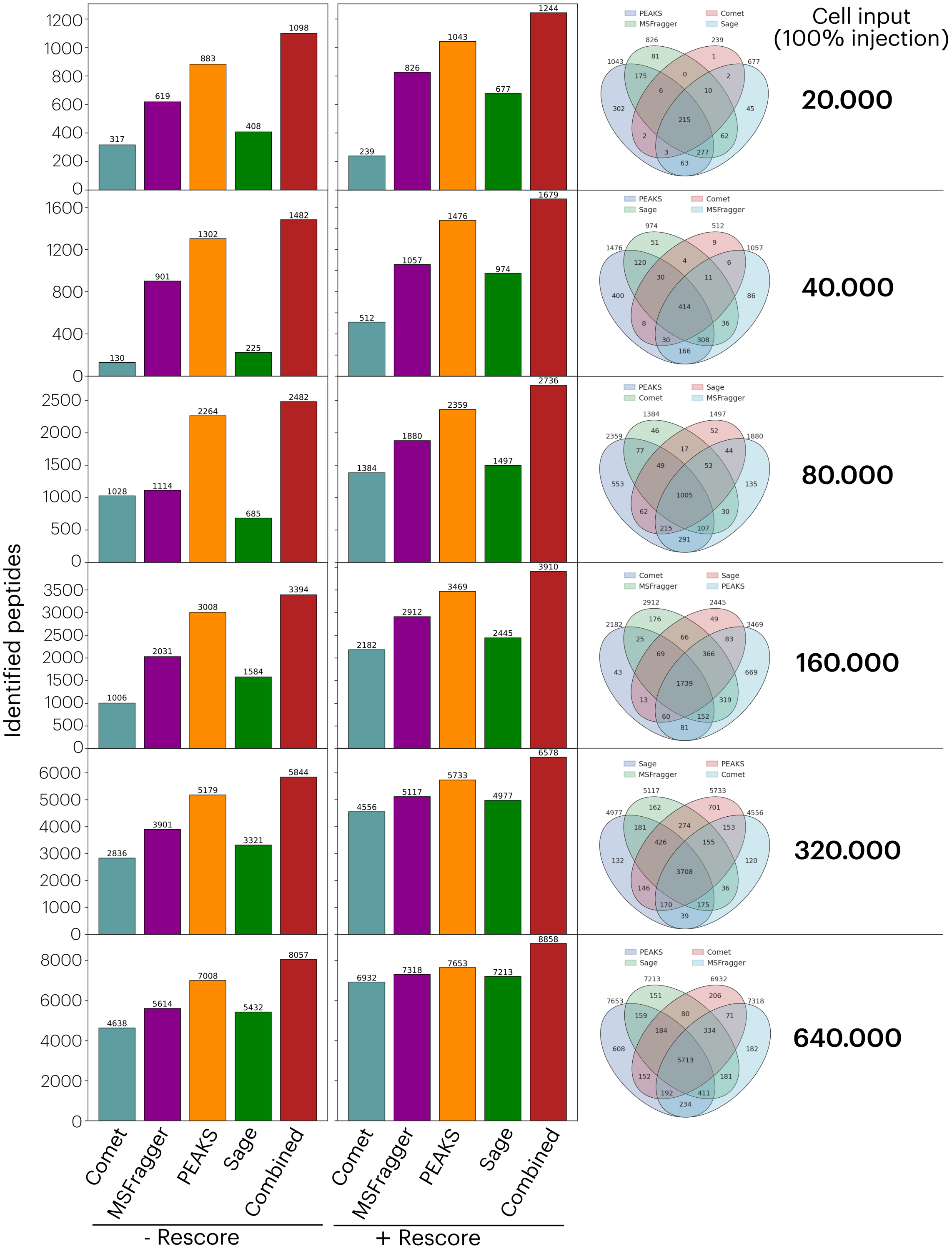

Supplemental Figure S13. **Identification boosting by multi-engine rescoring per JY cell input (ultrasensitive series).** (*Left*) The number of identified peptides per search engine or all four combined was shown prior and after data-driven rescoring by TIMS²Rescore (52). (*Right*) Venn diagram displaying the overlap of peptide sequences identified per search engine (after rescoring).

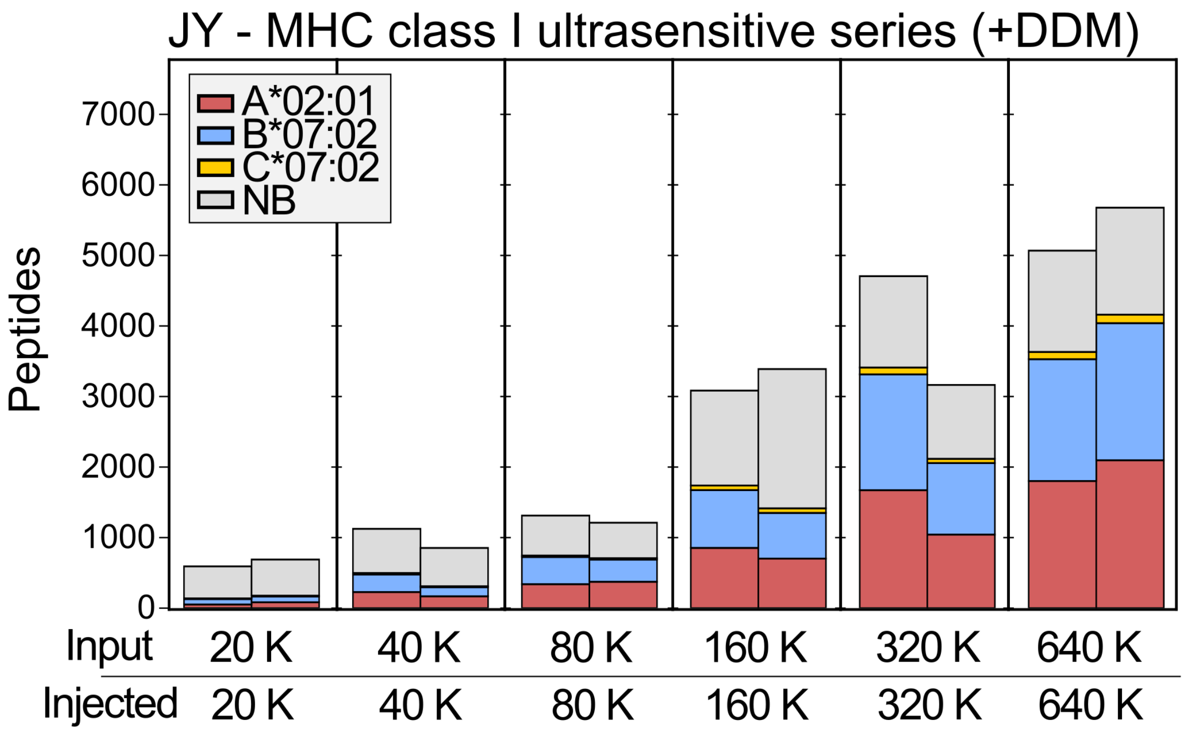

Supplemental Figure S14. Ultrasensitive immunopeptidomics in presence of n-dodecyl-β-D-maltoside. In an attempt to increase hydrophobic peptide recovery, a final volume of 0.02% n-dodecyl-β-D-maltoside (DDM) was added to each purified peptide mixture for 24 h prior to LC-MS/MS injection. Identified peptides sequences per sample were grouped per cell input amount from 640,000 (640 K) down to 20,000 (20 K) cells. Each cell input was processed and searched independently, and 100% of the input material was injected on timsTOF SCP. Peptides are colored according to binding prediction by netMHCpan-4.1 (53) to JY MHC class I alleles, indicating the predicted binders per allele (%Rank < 2, lowest if multiple alleles).

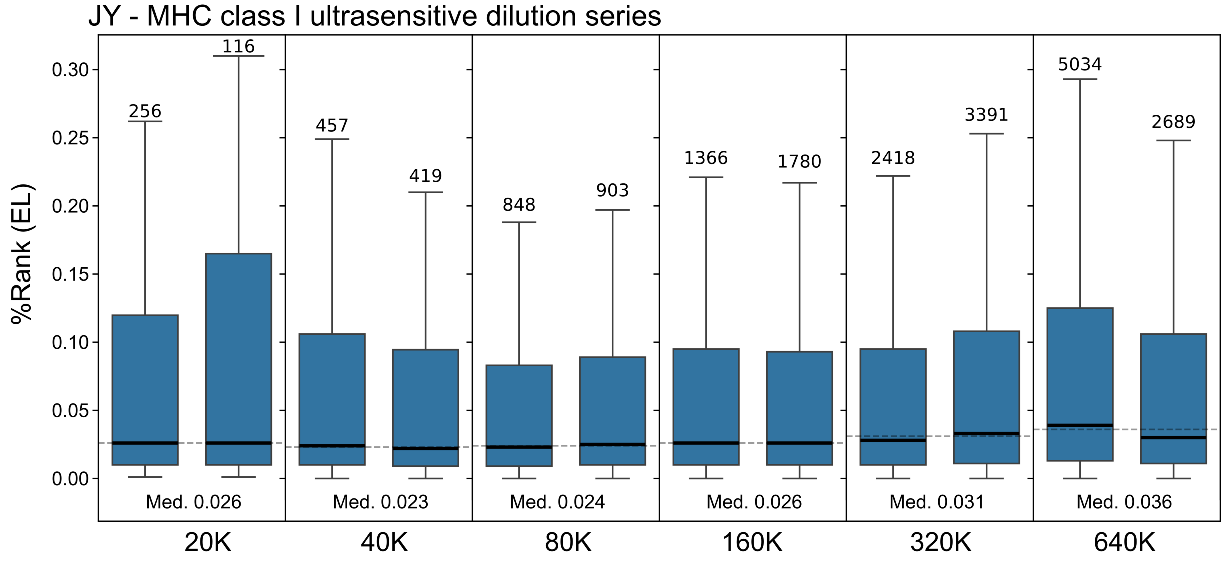

Supplemental Figure S15. Predicted binding strength for identified immunopeptides in the ultrasensitive JY cell input series. Boxplot distributions of the %Rank score calculated by NetMHCpan-4.1 (53) were shown for predicted binders (%Rank < 2) in each sample. Per cell input amount, the median %Rank was displayed for all predicted MHC binders.

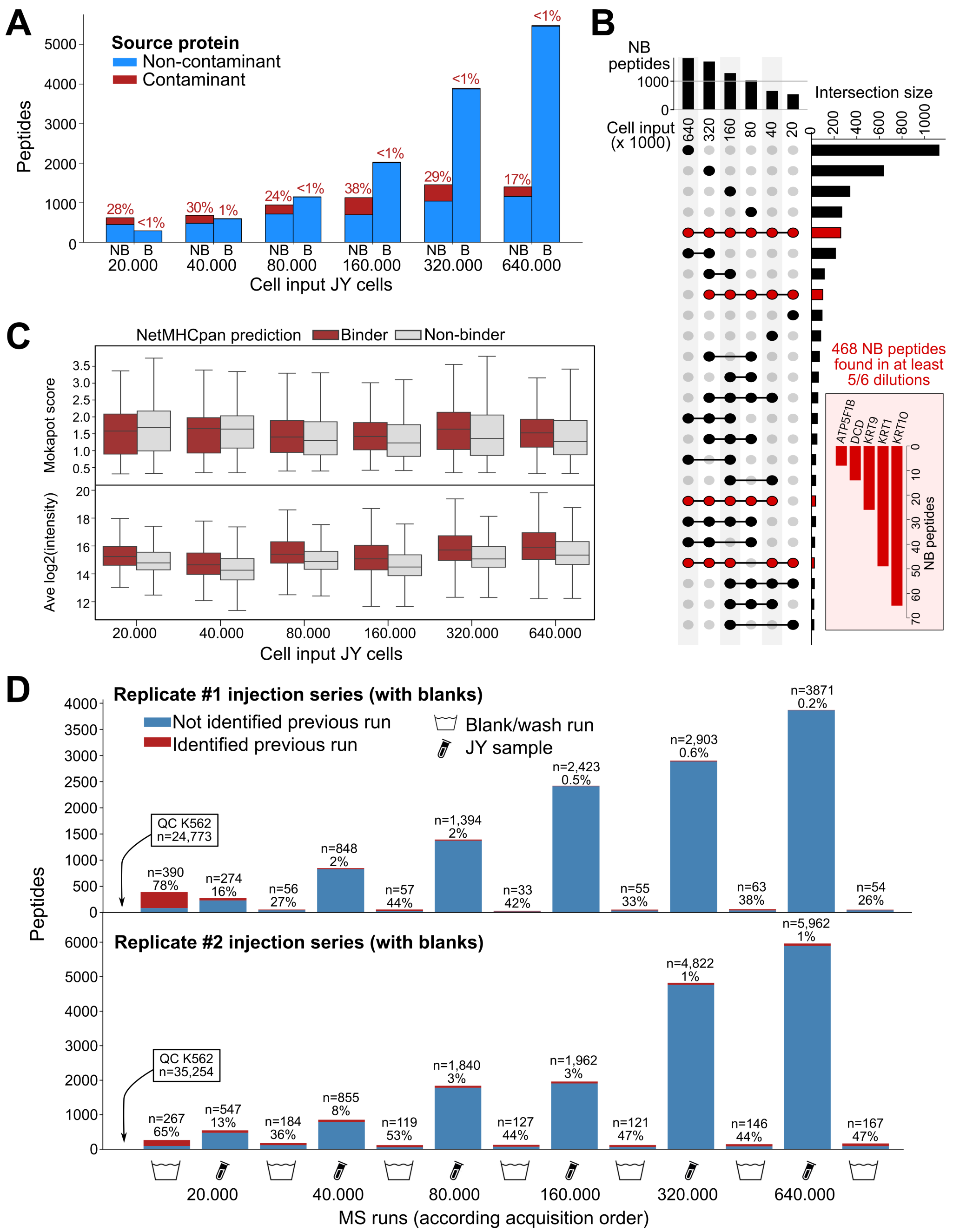

Supplemental Figure S16. Nonbinders in ultra-low cell input amounts are enriched in protein contaminants originating from sample preparation. (**A**) Per JY cell input the number of peptides identified from contaminant and non-contaminant source proteins (red and blue, respectively) was shown for NetMHCpan-4.1 (53) predicted binders (%Rank ≤ 2) and non-binders (B and NB, respectively). The MaxQuant contaminant database was used (246 proteins) to define contaminant source proteins. (**B**) UpSet plot showing the peptide overlap of NB peptides (only showing intersections ≥ 20 peptides). NB peptide intersections identified in at least five out of six cell inputs were shown in red, and the top five source proteins of these reproducible NBs were plotted (see inset). (**C**) Boxplots of the peptide mokapot score and average log2 intensity of NetMHCpan-4.1 predicted binders (red) and non-binders (grey) across the JY ultrasensitive cell input. (**D**) FragPipe peptide identification in JY cell input samples (sample symbol) and blank runs (wash symbol). All runs are ordered chronologically according to acquisition time (*left to right*). Peptides also identified in the preceding run, reflecting potential carry-over, were indicated in red, and their percentage indicated above the bar. Prior to the first blank run, a quality control (QC) of a K562 tryptic digest was analyzed in data-independent acquisition mode.

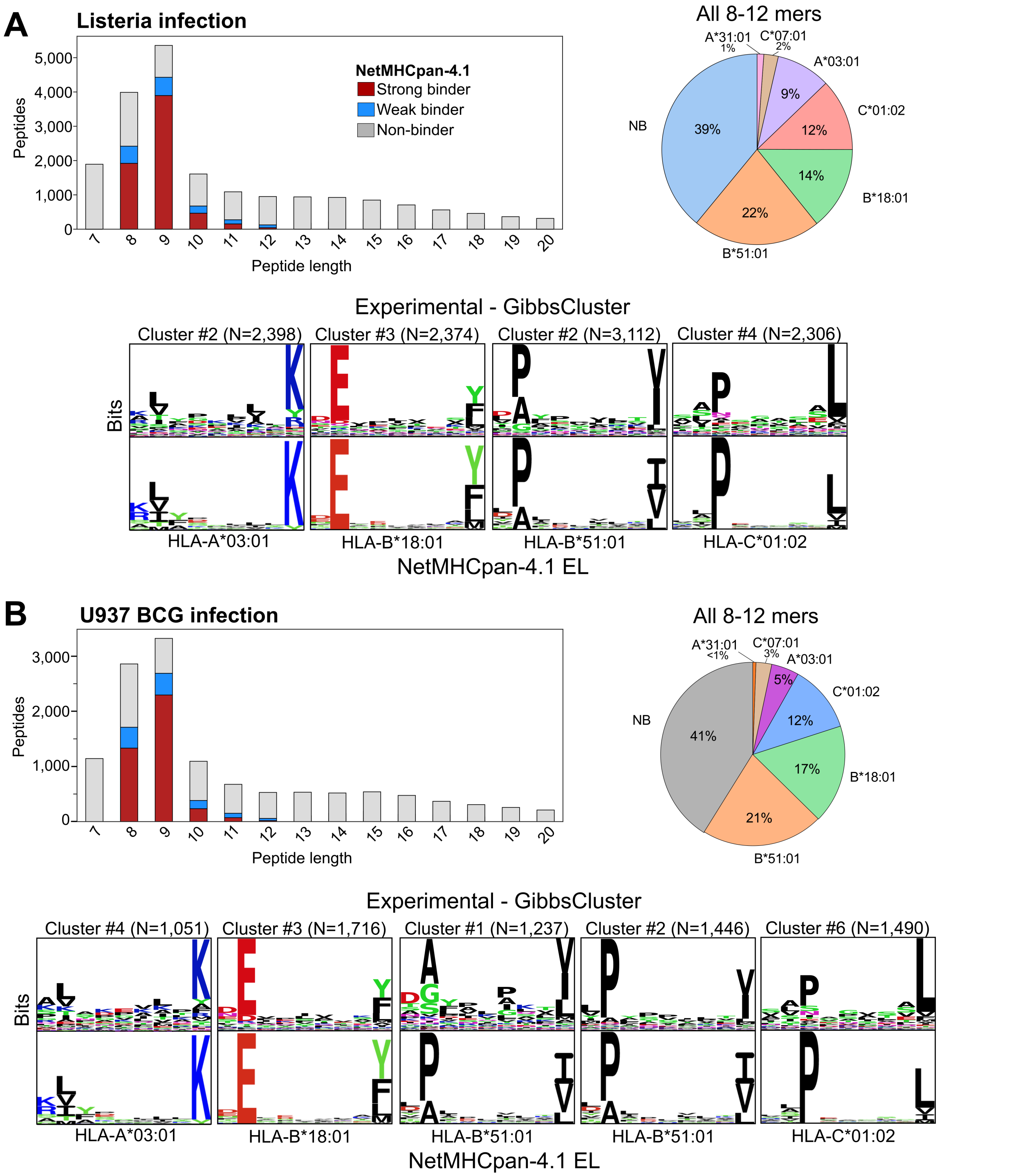

Supplemental Figure S17. Immunopeptidomics quality control of U937 cell cultures infected by *Listeria monocytogenes* (A) and *Mycobacterium bovis* BCG (B). A peptide length histogram showing the number of identified immunopeptides according to their NetMHCpan-4.1 (53) binding prediction, with strong binders (%Rank < 0.5) in red, weak binders (%Rank < 2) in blue, and non-binders (%Rank > 2) in grey. For all 8-12mer peptides, the proportion of best-binding HLA alleles was given in a pie chart. In addition, unsupervised GibbsCluster2.0 clustering (55) of 8-12mer peptides reveals sequence logos matching MHC class I allele eluted ligand (EL) motifs of netMHCpan-4.1 (53).

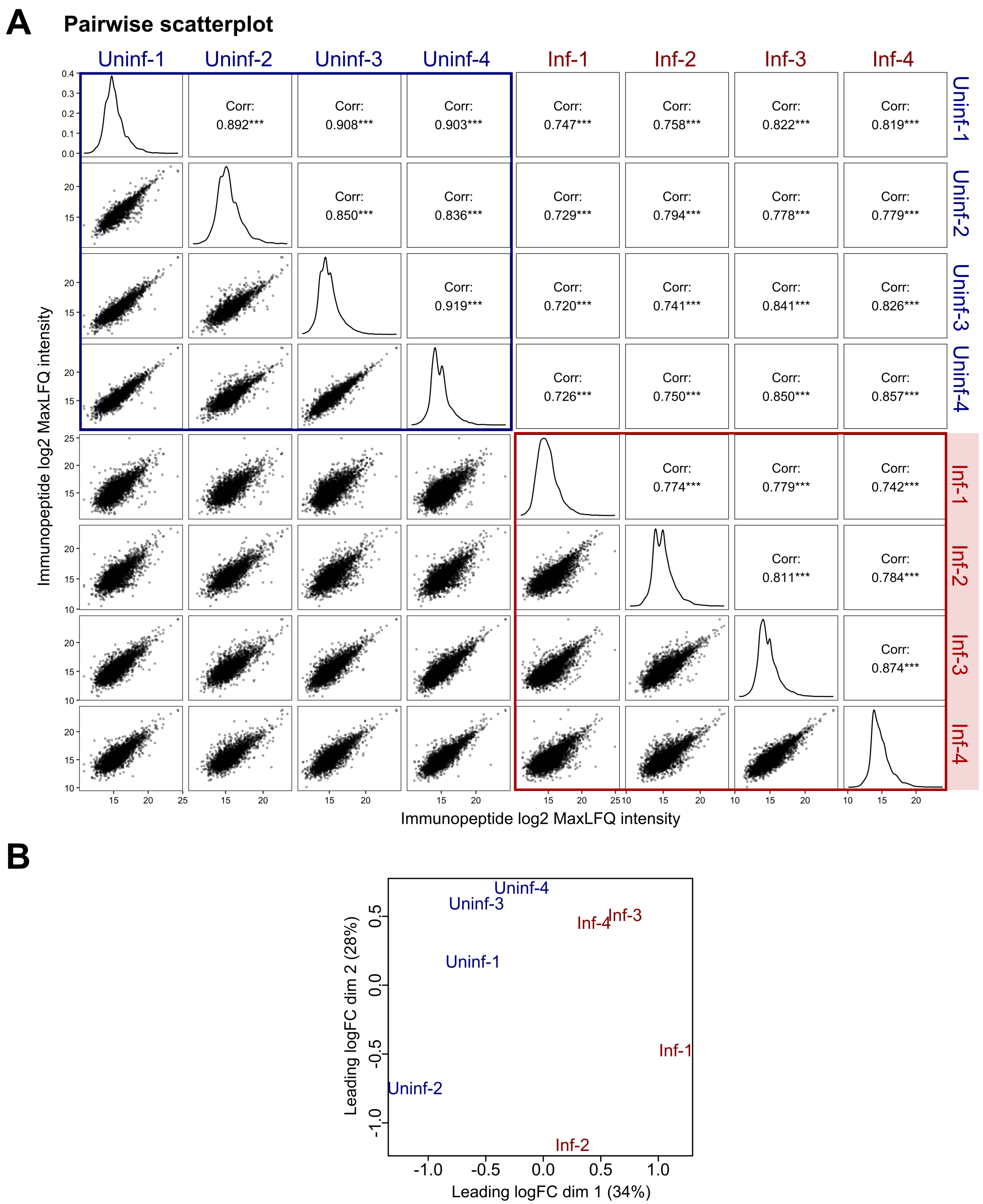

Supplemental Figure S18. Quantitative reproducibility and variation between BCG-infected and uninfected samples. (**A**) Pairwise scatter plots of log2-transformed MaxLFQ peptide intensities. The lower panels display pairwise scatter plots between samples, the upper panels show Pearson correlation coefficients, and the diagonal panels depict the distribution of intensities within each sample. Plots were made using the GGally (doi: 10.32614/CRAN.package.GGally) *ggpairs* function in R. Plots comparing uninfected ('Uninf') and BCG-infected ('Inf') samples were indicated in a blue and red rectangle, respectively. (**B**) Multidimensional scaling (MDS) plot of log2 normalized peptide intensities.

Supplemental Methods

This more detailed protocol explains the workflow for the generation of MHC I and -II immunoaffinity columns, and for the subsequent purification of MHC-I and MHC-II-presented immunopeptides from different cell types. This protocol is applicable to 32 million cells or less, and to a variety of tissues.

Prepare all the buffers and solutions using ultrapure water and LC-MS-grade reagents. Rinse the glass bottles three times with ultrapure water before preparing the solutions. Always use freshly prepared solutions.

1. Materials

1.1. Generation of immunoaffinity columns

1. Ultrapure water
2. Protein A - Sepharose® 4B Conjugate (#101041, Invitrogen) for W6/32 antibody & Protein G - Sepharose™ 4B beads (#101242, Invitrogen) for PdV5.2 antibody
3. 50 mM Tris-HCl 150 mM NaCl pH 8 (TBS)
4. Sodium tetraborate decahydrate (#B3545, Sigma-Aldrich)
5. Purified W6/32 anti-pan HLA-I antibody
6. Purified PdV5.2 anti-pan-HLA-II antibody
7. Dimethyl pimelimidate (DMP) crosslinker (#D8388, Sigma-Aldrich)
8. Ethanolamine (#149582500, Thermo Scientific)
9. TBS with 0.02% NaN3.
10. Reusable Econo glass columns (#7374150, Bio-Rad)
11. 15 mL Eppendorf Protein LoBind® tubes (#EP0030122216, Merck)
12. IKA roller 6 digital (#0004011000, IKA)
13. Gel electrophoresis system
14. Coomassie Brilliant blue stain (#20279, Thermo Scientific)

1.2. Isolation and purification of MHC-I and MHC-II binding peptides

1. Sodium deoxycholate (SDOC) (#1065040250, Millipore, Merck)
2. Octyl-beta D-glucopyranoside (OGP) (#O9882, Sigma-Aldrich)
3. 100 mM EDTA pH 8 (#EDS, Sigma-Aldrich)
4. Iodoacetamide (IAA) (#I1149, Sigma-Aldrich)
5. phenylmethylsulfonylfluoride (PMSF, #52332, Sigma-Aldrich).
6. Complete™ Mini, EDTA-free Protease Inhibitor mini tablets (#A32955, Pierce)
7. 50 mM Tris-HCl 150 mM NaCl pH 8 (TBS)
8. 100% acetonitrile (ACN)
9. 0.1% trifluoroacetic acid (TFA)
10. 25% ACN; 0.1% TFA
11. 40% ACN; 0.1% TFA
12. 10% Acetic acid
13. Resolvex A200 positive pressure processor (Tecan)
14. Filter microplate, 96-well, polypropylene, with 0.7 µm glass fiber membrane, 2 mL/well, long drip (#201007-100, Agilent)
15. Sep-Pak tC18 96-well Plate, 100 mg Sorbent per Well, 37 - 55 µm (#186002321, Waters)

16. 96-well polypropylene "Collection" plates, 2 mL per well (#201240-100, Agilent)
17. *Only for in vitro-infected samples*: Costar® Spin-X® Centrifuge Tube Filters, 0.22 µm Pore CA Membrane (#8161, Corning)
18. Optional: Pierce™ BCA Protein Assay Kit (#23225, Thermo Scientific)
19. 1.5 mL Eppendorf Safelock tubes
20. 1.5 mL Eppendorf Protein LoBind® tubes (#EP0030108116, Merck)
21. Bench-top centrifuge
22. SpeedVac vacuum concentrator

2. Methods

2.1. Generation of immunoaffinity columns for MHC Class I and II pull-down

1. Label two clean Econo columns as "W6/32-protein A" and "PdV5.2-protein G". Add 2 mL of freshly resuspended beads per column.
2. Wash the beads 3 times with 10 mL TBS. Allow all buffer to drain before closing the column with the bottom cap.
3. Per column, dilute 3 mg W6/32 anti-MHC-I antibody or 1.5 mg PdV5.2 anti-MHC-II antibody in 5 mL TBS. Take a 60 µL aliquot of each antibody solution for quality control (QC1, see section 2.2) before adding to the protein A and protein G beads, respectively.
4. Incubate 1 h at room temperature (RT) using an IKA roller at 80 rpm.
5. While incubating, prepare 0.2 M sodium tetraborate, pH 9 and thaw the DMP crosslinker. Make sure the crosslinker is at room temperature before opening the vial.
6. After incubation, collect the unbound antibody solution in a 15 mL tube. Take a 60 µL aliquot of the unbound antibody solution for quality control (QC2).
7. Wash the columns 5 times with 10 mL of 0.2 M sodium tetraborate, pH 9.
8. Resuspend the beads in 5 mL 0.2 M sodium tetraborate, pH 9 and take a 60 µL aliquot for quality control (QC3).
9. Per column, dissolve 26 mg dimethyl pimelimidate (DMP) crosslinker in 5 mL sodium tetraborate and immediately add it to the column. Close the column and incubate for 45 mins at RT on an IKA roller (80 rpm). Do not incubate shorter or longer to avoid under- or over-crosslinking, respectively.
10. While waiting, prepare 0.2M ethanolamine by dissolving 6 mL ethanolamine solution in 500 mL TBS. Adjust to pH 8 with 37% HCl.
11. Remove the column caps and allow the liquid to drain. Wash the beads 3 times with 10 mL 0.2 M sodium tetraborate.
12. Resuspend the beads in 5 mL of 0.2 M sodium tetraborate and take an aliquot of 60 µL for quality control (QC4).
13. Remove the caps and allow the liquid to drain. Wash the beads 3 times with 10 mL of 0.2 M ethanolamine solution.
14. Close the column with the bottom cap and add 5 mL of 0.2 M ethanolamine solution. Rotate at RT for 2 h on an IKA roller (80 rpm).
15. Remove the column caps and allow the ethanolamine to drain. Wash the beads 5 times with 10 mL TBS.
16. Transfer the beads to 15 mL Protein LoBind Tubes and let them settle overnight.

17. The next day, remove the supernatant and add 1 mL TBS with 0.02% (V/V) NaN_3 per mL of settled beads. Store up to 3 months at 4°C.

2.2. Quality control of the immunoaffinity columns for MHC Class I and II pull-down

1. Add 15 µL 4x Laemmli buffer to all quality control samples (QC1-QC4) and boil 5 minutes at 95°C.
2. Load 30 µL of each QC sample on a 4-15% gradient polyacrylamide gel and perform the electrophoresis at 140V.
3. Briefly wash the gel in purified water before staining with Coomassie brilliant blue for 30-60 minutes. Optionally silver staining can also be used.
4. Wash the gel two times briefly and one time for 15 minutes in purified water before imaging.
5. The antibody heavy chain (50 kDa) and light chain (25 kDa) should be clearly detectable in QC1 (antibody input) and QC3 (beads with conjugated antibody, not crosslinked). There should only be a faint signal in QC2 (unconjugated antibody), and no signal in QC4 (antibody crosslinked to the beads).

2.3. High-throughput isolation and purification of MHC-I and MHC-II peptides

This procedure is performed in a semi-automated manner in 96-well format, using a Tecan Resolvex A200 positive pressure processor.

2.3.1. Lysate preparation for the purification of MHC-I and MHC-II peptides

When processing infected samples, perform the lysis step in a flow cabinet in the appropriate biosafety level facility.

1. Prepare the lysis buffer, consisting of 0.25% sodium deoxycholate (SDOC), 1% Octyl-beta D-glucopyranoside (OGP), 1 mM EDTA, 0.2 mM Iodoacetamide (IAA), 1 mM phenylmethylsulfonylfluoride and 1.25x complete™ Mini, EDTA-free Protease Inhibitor in TBS pH 8. Prepare fresh and keep on ice.
2. Take out the cell pellets from -80 °C and thaw them on ice.
3. Add 0.1 mL cold lysis buffer to each pellet and lyse the cells on ice for 1 h, pipetting up and down 10X every 15 minutes. Use 50 µL lysis buffer when processing 10^6 cells or less.
4. Clear the lysate by centrifuging at 20,000g at 4°C for 10 min (4°C). Transfer the supernatant to a fresh tube and centrifuge at 20,000g for 30 minutes.
5. When processing *in vitro* infected samples, transfer the supernatant to a 0.22 µm Spin-X Centrifuge Tube Filter and centrifuge 5 minutes at 16,000g at 4°C.
6. Transfer the clear lysate to a fresh tube and keep on ice.
7. For quantitative immunopeptidomics analysis, measure and normalize the protein concentrations using BCA protein assays.
8. Optionally, take 3% of the lysate ('input') for immunoblot analysis.

2.3.2. Preparation of 96-well plates for IP

1. Label two 96-well filter plates with "MHC-I" and "MHC-II".

2. Mark the wells that will be used and seal the remaining unused wells with the microplate sealing film.
3. Rinse the Resolvex A200 solvent lines with ultrapure water before connecting them to the correct buffer recipients. Indicate in the Resolvex A200 software which wells will be used.
4. Using the Resolvex A200, pre-wash the wells twice with 1 mL 100% ACN, twice with 1 mL 0.1% TFA and three times with 1 mL TBS. After each wash, apply 10% low positive pressure for 1-2 minutes to drain the liquid from the wells.
5. Load 0.2 mL resuspended cross-linked immunoaffinity beads per well and wash 5 times with 1 mL TBS. Make sure the wells are completely dry before proceeding to the next step.

2.3.3. Isolation of MHC-I and MHC-II peptide complexes

1. Load 0.1 mL clear lysate on the MHC-I filter plate. Stack the filter plate on top of a clean collection plate and incubate 1 h at 4 °C.
2. Using the Resolvex A200, elute the lysate from the MHC-I filter plate to the collection plates by applying 10% low pressure for 1 minute. Optionally, take 3% of the collected lysate to evaluate MHC-I depletion via immunoblot analysis ('MHC-I-depleted').
3. Transfer the collected lysate to the MHC-II filter plate and incubate the plate at 4 °C for 1 h. Optionally the flowthrough of the MHC-I filter plate can be directly collected in the MHC-II filter plate to minimize sample losses.
4. Meanwhile, wash the MHC-I plate five times with 1 mL TBS.
5. After incubation, stack the MHC-II filter plate on top of a fresh collection plate and elute the lysate by applying 10% low pressure (coarse: 5%-10%). Transfer the flow-through to Eppendorf tubes, and, optionally, take 3% of the lysate to evaluate MHC-II depletion via immunoblot analysis ('MHC-II-depleted'). Store the rest of flow-through at -80 °C.
6. Wash the MHC-II filter plate five times with 150 mM NaCl in 50 mM Tris-HC (pH 8).

2.3.4. Elution and purification of MHC-I and MHC-II-bound peptides

1. Label two Sep-Pak tC-18 96-well plates with "MHC-I" and "MHC-II". Using the Resolvex A200, pre-wash the wells twice with 1 mL 100% ACN and 3 times with 1 mL 0.1% TFA. Apply 1-3% high positive pressure to drain the liquid from the wells.
2. Mount each MHC filter plate on a Sep-Pak plate and elute the MHC-I- and MHC-II-peptide complexes with five times 0.2 mL 10% acetic acid. With each elution step, incubate 5 minutes at RT before applying 10% low positive pressure.
3. Remove the filter plates and continue with the Sep-Pak plates.
4. Apply pressure to remove all acetic acid before washing each Sep-Pak plate three times with 1 mL 0.1% TFA.
5. Place the Sep-Pak plate on top of a clean collection plate. Elute the MHC-I peptides with three times 500 µL 25% ACN 0.1% TFA, and the MHC-II peptides with 40% ACN 0.1% TFA.

6. Transfer the eluted peptides to pre-labeled 1.5 mL protein LoBind tubes and partially dry the peptides before transferring to LC-MS autosampler vials. Store the dried peptides at -20°C until LC-MS/MS analysis.
7. Resuspend the dried peptides in 50 μL 0.1% TFA for 25% injection, or in 17 μL for 100% injection. Vortex and centrifuge briefly.
